# Antihypertensive effects of exercise involve reshaping of gut microbiota and improvement of gut-brain axis in spontaneously hypertensive rat

**DOI:** 10.1080/19490976.2020.1854642

**Published:** 2020-12-31

**Authors:** Wen-Jie Xia, Meng-Lu Xu, Xiao-Jing Yu, Meng-Meng Du, Xu-Hui Li, Tao Yang, Lu Li, Ying Li, Kai B. Kang, Qing Su, Jia-Xi Xu, Xiao-Lian Shi, Xiao-Min Wang, Hong-Bao Li, Yu-Ming Kang

**Affiliations:** a Department of Physiology and Pathophysiology, Xi’an Jiaotong University School of Basic Medical Sciences, Shaanxi Engineering and Research Center of Vaccine, Key Laboratory of Environment and Genes Related to Diseases of Education Ministry of China, Xi’an China; b Department of Nephrology, The First Affiliated Hospital of Xi’an Medical University, Xi’an China; c Center for Neuron and Disease, Frontier Institutes of Science and Technology, Xi’an Jiaotong University, Xi’an China; d Microbiome Consortium and Center for Hypertension and Precision Medicine, Department of Physiology and Pharmacology, College of Medicine and Life Sciences, University of Toledo, Toledo, OH USA; e Department of Ophthalmology and Visual Sciences, University of Illinois at Chicago, Chicago, IL, USA; f Department of Pharmacology, School of Basic Medical Sciences, Xi’an Jiaotong University Health Science Center, Xi’an China

**Keywords:** Exercise, hypertension, microbiota, gut-brain axis, microglia

## Abstract

Exercise (Ex) has long been recognized to produce beneficial effects on hypertension (HTN). This coupled with evidence of gut dysbiosis and an impaired gut-brain axis led us to hypothesize that reshaping of gut microbiota and improvement in impaired gut-brain axis would, in part, be associated with beneficial influence of exercise. Male spontaneously hypertensive rats (SHR) and Wistar Kyoto (WKY) rats were randomized into sedentary, trained, and detrained groups. Trained rats underwent moderate-intensity exercise for 12 weeks, whereas, detrained groups underwent 8 weeks of moderate-intensity exercise followed by 4 weeks of detraining. Fecal microbiota, gut pathology, intestinal inflammation, and permeability, brain microglia and neuroinflammation were analyzed. We observed that exercise training resulted in a persistent decrease in systolic blood pressure in the SHR. This was associated with increase in microbial α diversity, altered β diversity, and enrichment of beneficial bacterial genera. Furthermore, decrease in the number of activated microglia, neuroinflammation in the hypothalamic paraventricular nucleus, improved gut pathology, inflammation, and permeability were also observed in the SHR following exercise. Interestingly, short-term detraining did not abolish these exercise-mediated improvements. Finally, fecal microbiota transplantation from exercised SHR into sedentary SHR resulted in attenuated SBP and an improved gut-brain axis. These observations support our concept that an impaired gut-brain axis is linked to HTN and exercise ameliorates this impairment to induce antihypertensive effects.

## Introduction

Accumulating evidence has implicated altered gut microbiota in hypertension (HTN).^1,2^ In addition to demonstration of dysbiosis, fecal microbiota transplantation (FMT) from spontaneously hypertensive rats (SHR) into Wister Kyoto normotensive rats (WKY) or hypertensive human donors into germ-free mice resulting in elevated blood pressure (BP) and sympathetic activity,^3–5^ are persuasive evidence for a direct influence of gut microbiota on the BP regulation. There is a surge in evidence of a dysfunctional gut-brain axis in HTN, including an increase in neural trafficking between the gut and autonomic brain regions,^6^ the total number and activated microglia and neuroinflammation,^7^ the sympathetic nerve activity in the gut and intestinal tyrosine hydroxylase and norepinephrine levels.^5,6^

Exercise is an established non-pharmacological approach for the treatment and control of HTN in animals and humans.^8–10^ Moderate-intensity aerobic activity has been recommended as a therapy for lowering BP according to the current American Heart Association guidelines.^11^ Previous studies have demonstrated a sustained reduction in BP, amelioration of cardiac hypertrophy and diastolic function in angiotensin II–induced hypertensive rats after short-term detraining.^12^ These beneficial effects were associated with reduced pro-inflammatory cytokines and attenuated oxidative stress within the brain of the hypertensive rats.^12–14^ Recent evidence suggests that moderate-intensity exercise improves functional activation of the brain^15^ and influences the gut by increasing the diversity and abundance of gut microbiota in metabolic diseases such as diabetes, obesity and HTN.^16–19^ Given the importance of exercise in the treatment and control of HTN and the involvement of gut microbiota and dysfunctional gut-brain axis in HTN, we tested the hypothesis that exercise would reshape gut microbiota, ameliorate dysfunctional gut-brain axis and would contribute to improvement in HTN pathophysiology. Indeed, our results demonstrated that moderate-intensity exercise training, even if short-term detraining, produces a long-term antihypertensive effect with profound changes in gut microbiota, improvement in gut pathology and permeability, and rebalanced dysfunctional gut-brain axis in hypertensive rats.

To test if the changes in gut microbiota composition were involved in the antihypertensive effects induced by sustained exercise training, we performed FMT from exercised SHR into sedentary SHR by oral gavage. We found that FMT intervention significantly lowered the systolic BP and changed the gut microbiota composition in SHR.

## Materials and Methods

### Animal and experimental design

This research was designed according to the National Institutes of Health (NIH) Guide for the Care and Use of Laboratory Animals, and approved by the Ethics Committee of Laboratory Animals of Xi’an Jiaotong University. Twelve-week-old male Wistar Kyoto (WKY) rats and SHR were purchased from the Charles River Laboratories (Beijing, China) and allowed to acclimate for 2 weeks before moderate-intensity exercise. The rats were housed in a temperature- and humidity-controlled room with a 12-h/12-h light-dark cycle. All the rats were fed standard chow and tap water *ad libitum*.

**Figure 1.**
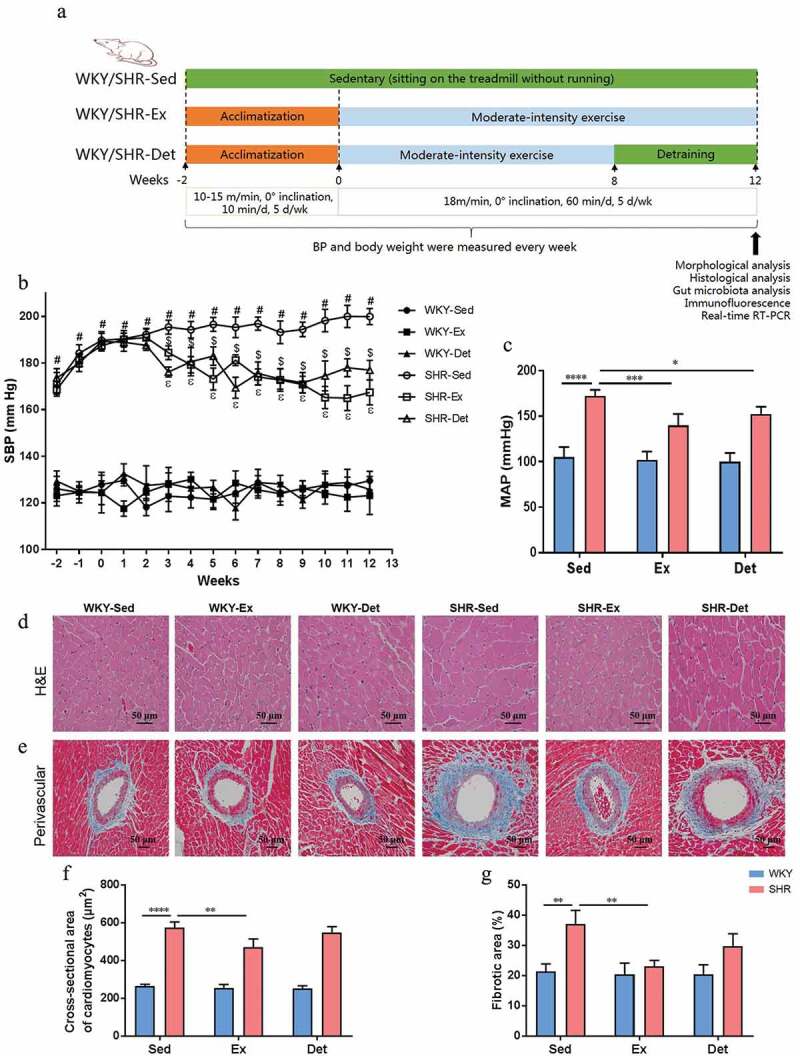
Effects of exercise training and detraining on blood pressure (BP) and morphological changes in organs in SHR and WKY rats. (a) Schematic illustration of exercise training and detraining program. (b) Time course of systolic BP (SBP) was measured via tail-cuff method in normotensive and hypertensive rats. (c) At the end of the study, mean arterial BP (MAP) was measured by intra-arterial recording into left carotid artery. (d) Representative micrographs showing the results of hematoxylin-eosin (H&E) staining in terms of cardiomyocytes size in different groups. (e) Representative micrographs of Masson’s trichrome staining assay showing perivascular fibrosis in myocardium. (f) A column diagram showing quantitative analysis of cross-sectional area of cardiomyocytes. (g) A column diagram showing quantitative analysis of the relative fibrotic area. n = 8–10 rats per group. Data are presented as mean ± SEM. SBP data were analyzed by one-way repeated-measures ANOVA with a Tukey’s post-hoc test. ^#^*P*< .05 SHR-Sed versus WKY-Sed; ^$^*P*< .05 SHR-Sed versus SHR-Ex; ^ε^*P*<.05 SHR-Sed versus SHR-Det. Other parameters were analyzed by two-way ANOVA with a Tukey’s post-hoc test. *P< .05; **P< .01; ***P< .001; ****P< .0001

*Experiment 1*: Animals were randomly assigned to six different groups of 8–10 animals each: WKY + Sedentary (WKY-Sed), WKY + Exercise (WKY-Ex), WKY + Detraining (WKY-Det), SHR + Sedentary (SHR-Sed), SHR + Exercise (SHR-Ex), and SHR + Detraining (SHR-Det). Trained rats underwent moderate-intensity exercise for 12 weeks, whereas detrained groups underwent 8 weeks of exercise followed by 4 weeks of detraining. Systolic blood pressure (SBP) was measured every week using tail-cuff plethysmography at room temperature in conscious animals. For each group, the fecal and small intestine samples were collected for analyses of the gut microbiota, gut inﬂammation, and tight junction protein expression. The brain was harvested and evaluated for changes in microglia and neuroinflammation in autonomic regions. Figure 1a shows an outline of the protocol.

*Experiment 2*: To investigate the potential mechanistic link between the gut microbiota and sustained exercise-induced effects in hypertension, we examined the effects of gut microbiota using FMT from exercise-improved hypertensive rat donors to SHR. Fresh fecal contents were collected from experimental groups and pooled. Twelve-week-old recipient SHR were orally gavaged with donor fecal contents for five consecutive days, and once in every 2 days for 4 weeks. Recipient SHR were randomly assigned to five different groups of 8–11 animals each: SHR with phosphate buffer saline (S-PBS), SHR with WKY-Sed microbiota (S-W-Sed), SHR with SHR-Sed microbiota (S-S-Sed), SHR with SHR-Ex microbiota (S-S-Ex), and SHR with SHR-Det microbiota (S-S-Det). The BP from each group was measured using the tail-cuff occlusion and acute experiment method. The composition of gut microbiota and changes of microglia in autonomic regions were examined as described in experiment 1.

### Exercise and detraining protocol

All rats were acclimated to a motorized treadmill (FT-200, Taimen Co., Cheng du, China) for 2 weeks. After acclimation, rats in the exercise groups (WKY-Ex and SHR-Ex) performed moderate-intensity exercise (5 days per week; 60 min per day, 0° inclination) for 12 weeks. Rats in the detraining groups (WKY-Det and SHR-Det) were given 8 weeks of moderate-intensity exercise followed by 4 sedentary weeks. The training intensity was determined and maintained as previously described.^12^

Briefly, training intensity was set at approximately 60% of the maximal aerobic velocity (MAV), which corresponds to moderate intensity (18–20 m/min). This training intensity was maintained throughout the study period. The MAV was evaluated from an incremental exercise test reported previously.^12,13^ The rats in sedentary groups (WKY-Sed and SHR-Sed) were placed on an unmoving treadmill during the training duration.

### Fecal microbiota transplantation (FMT)

Fecal microbiota transplantation in recipient rats was carried out as previously reported with several modifications.^4,20^ Briefly, stool contents were harvested and pooled from WKY-Sed, SHR-Sed, SHR-Ex, and SHR-Det groups in experiment 1. Stool contents were diluted 1:20 in sterile PBS and centrifuged at 1000 rpm for 15 min. The supernatant was aliquoted and stored at −80°C for further use. Recipient SHR was orally gavaged with 1 mL ceftriaxone sodium (400 mg/kg/day) every two days for two weeks. After two weeks of antibiotic treatment, recipient rats were orally gavaged with donor fecal contents (1 mL) as explained above.

### Blood pressure measurements

Two methods were used to measure BP. Tail-cuff plethysmography was carried out weekly in conscious animals as previously reported.^4,21^ A minimum of 12 consecutive pressure readings were averaged for each rat. Additionally, the left carotid artery of the isoflurane-anesthetized rat was cannulated with polyethylene catheters to measure direct BP as previously described.^4,22^ The catheters with heparinized saline (100 units/ml) were connected to a pressure transducer attached to computer software (NIBP, AD Instruments, Australia). Mean arterial pressure (MAP) and heart rate (HR) data were collected during the last 30 min and averaged.

### Brain slice electrophysiology

Coronal hypothalamic slices, including the paraventricular nucleus (PVN) were obtained using standard procedures as previously described.^23,24^ Briefly, rats were decapitated after 1–2% isoflurane anesthesia. The whole brain was removed and submerged in ice-cold oxygenated (95% O_2_ and 5% CO_2_) artificial cerebrospinal fluid (ACSF) containing 252 mM sucrose, 2.5 mM KCl, 6 mM MgSO_4_, 0.5 mM CaCl_2_, 25 mM NaHCO_3_, 1.2 mM NaH_2_PO_4_, 10 mM glucose, and pH 7.4. Then the brain slices (300 µm) were cut with a Vibratome slicer (VT1200S, Leica, Wetzlar, Germany) and transferred to a perfusion chamber with fresh ACSF containing 124 mM NaCl, 4.4 mM KCl, 2 mM CaCl_2_, 1 mM MgSO_4_, 25 mM NaHCO_3_, 1 mM NaH_2_PO_4_, 10 mM glucose, and pH 7.4 at 37°C for 1 h.

Whole-cell voltage-clamp recordings were performed on PVN neurons within hypothalamic slices and visualized under an upright microscope (BX51W, Olympus, USA) equipped with differential interference contrast (DIC) optics. In the recordings of spontaneous excitatory postsynaptic currents (sEPSCs), the patch electrodes (3–5 MΩ) were filled with intracellular solution containing 145 mM K-gluconate, 5 mM NaCl, 1 mM MgCl2, 0.2 mM ethylene glycol-bis (β-aminoethyl ether)-N,N,N′,N′-tetraacetic acid (EGTA), 10 mM N-2-hydroxyethyl piperazine-N′-2-ethanesulfonic acid (HEPES), 2 mM Mg-ATP, 0.1 mM Na-GTP, and pH 7.2. The change of membrane potential was detected using a Multiclamp 700B Amplifier and filtered using a Digidata 1440A digital acquisition system (Axon Instruments).

### Tissue collection, left ventricular hypertrophy, and remodeling

At the end of the experiment, rats were euthanized with isoflurane. The blood samples were harvested from the abdominal aorta, quickly separated, and stored at −80°C. The heart and brain tissues were rapidly removed and cleaned, and the left ventricles (LV) were separated and weighed. The ratios of heart weight (HW)/body weight (BW), HW/tibia length (TL), and LV weight (LVW)/TL were calculated. The PVN tissues were cut and isolated from frozen brain tissues as previously described.^25–27^ All tissues were stored at −80°C until further molecular analysis or fixed in formalin in phosphate-buffered saline (PBS) at 4°C for histological and immunofluorescence analyses.

Further, a representative image of cardiomyocyte hypertrophy and perivascular fibrosis in the left ventricle was evaluated histologically in paraffin-embedded 5 µm cross-sections stained with hematoxylin-eosin (H&E) and Masson-trichrome staining, respectively.^28^

### Histological evaluation of gut pathologies

The general morphology and collagen formation of the ileum and proximal colon samples were evaluated as previously described.^6,21^ Briefly, paraffin-embedded 5 µm sections were stained with H&E and Masson-trichrome to visualize the general morphology and fibrosis. The fibrosis, thickness of the smooth muscle cell layer, crypt depth, villi length, number of goblet cells per 100 epithelial cells, and the ratio of goblet cells/villi were quantified using Image J software (National Institutes of Health, Bethesda, MA, USA, 2012).

### Real-time RT-PCR analysis

Total RNA was extracted from the ileum (small intestine) with TRIzol reagent (Invitrogen Corporation, USA) and converted to cDNA according to the manufacturer’s protocols. The mRNA concentration in the samples was measured spectrophotometrically and purity of RNA checked by 260/280 ratio. A quantitative real-time PCR was carried out using the Mx3005P Detection System (Agilent Technologies, USA). The sequences of the sense and antisense primers used for amplification are listed in Table 1. The gene expression fold change was normalized to the control sample and processed by the 2 – ^ΔΔCT^ method using GAPDH as an internal control. We did not observe significant expression differences in GAPDH between samples treated with exercise and detraining.

**Table 1.** Rat primers used for real-time RT-PCR

Rat genes	Forward Primer (5′-3′)	Reverse Primer (5′-3′)
TNF-α	GTCGTAGCAAACCACCAAGC	TGTGGGTGAGGAGCACATAG
IL-1β	GCAATGGTCGGGACATAGTT	AGACCTGACTTGGCAGAGA
IL-6	TCTGGTCTTCTGGAGTTCCGT	GCATTGGAAGTTGGGGTAGGA
TLR2	GCACTTGAGCGAGTCTGCTTTC	GAACAAATAGAACTGGGGGATGTG
TLR4	GGCTGTGGAGACAAAAATGACCTC	AGGCTTGGGCTTGAATGGAGTC
Tjp1	AGCGAAGCCACCTGAAGATA	GATGGCCAGCAGGAATATGT
Ocln	CTGTCTATGCTCGTCATCG	CATTCCCGATCTAATGACGC
Cldn4	CGAGCCCTGATGGTCATCAG	CGGAGTACTTGGCGGAGTAG
HMGB1	CTAGCCCTGTCCTGGTGGTATT	CCAATTTACAACCCCCAGACTGT
TH	CGAGCTGTGAAGGTGTTTGA	GTACACCTGGTCCGAGAAGC
GAPDH	AGACAGCCGCATCTTCTTGT	CTTGCCGTGGGTAGAGTCAT

TNF-α, tumor necrosis factor-alpha; IL, interleukin; TLR, toll-like receptor; Tjp1, tight junction protein 1; Ocln, occluding; Cldn4, claudin 4; HMGB1, high mobility box 1; TH, tyrosine hydroxylase; GAPDH, Glyceraldehyde 3-phosphate dehydrogenase.

Analysis of pro-inﬂammatory cytokines the mRNA levels in the PVN followed the same workflow outlined above with the following differences. We dissected and harvested the hypothalamic tissue, including PVN from freshly frozen brains as previously described.^25,26^ Briefly, a coronal section was cut from −0.90 to −2.15 mm posterior to bregma. These sections were mounted on slides and the PVN was isolated using a brain punch (Stoelting Co., Wood Dale, IL) for mRNA extraction.

### Immunohistochemistry and immunofluorescence staining

Brains and intestine samples were excised and post-fixed in 4% paraformaldehyde in PBS for 24 h. They were placed in 30% sucrose solution for 48 h, and used to prepare blocks in frozen OCT or paraffin. Brain (18 μm) and gut (5 μm) sections were washed with PBS containing 0.3% Triton X-100 for 15 min and blocked for 40 min in 10% goat serum in PBS. The expression of ionized-binding adaptor molecule 1 (Iba1) and NeuN in the PVN and tyrosine hydroxylase (TH) in the proximal colon was evaluated by immunofluorescence. The brain sections were incubated with a rabbit anti-Iba1 primary antibody (1:400; Wako 079–19741) for microglia and a mouse anti-NeuN primary antibody (1:300; Millipore, Massachusetts, MA) for neurons at 4°C overnight, followed by incubation with an Alexa fluor 488-labeled goat anti-rabbit secondary antibody (1:1000; Invitrogen, Carlsbad, CA) and Alexa fluor 594-labeled goat anti-mouse secondary antibody (1:1000; Invitrogen, Carlsbad, CA) for 1 h at room temperature. The intestinal sections were incubated with a rabbit anti-TH primary antibody (1:400; AB152, Millipore Sigma, Burlington, MA) at 4°C overnight, followed by incubation with a FITC-labeled goat anti-rabbit secondary antibody (1:500; Invitrogen, Carlsbad, CA) for 1 h at room temperature. The sections were observed with a confocal laser-scanning microscope (Lycra, Germany). Immunoreactivity of Iba1 (green), NeuN (red) and DAPI (blue) was pictured. ImageJ software was used to quantify the total number and the proportion of activated microglia within a 200 × 200 µm^2^ area of PVN. The percentage of TH-positive areas was determined by counting 10 random fields per section under a microscope and quantified by Image J software. The expression of tight junction proteins including occluding (Ocln), tight junction protein 1 (Tjp1) and claudin 4 (Cldn4) in the small intestine (ileum) was assessed by immunohistochemistry. Deparaffinized sections were incubated with primary antibodies rabbit anti-Ocln (1:400; Abcam, Cambridge, USA), rabbit anti-Tjp1 (1:200; Invitrogen, Carlsbad, CA), and rabbit anti-Cldn4 (1:400; Abcam, Cambridge, USA) overnight at 4°C. After washing with PBS, sections were incubated with the secondary antibody (1:300; ABC staining system kit, Santa Cruz, CA, USA) labeled with horseradish peroxidase (HRP) for 1 h at room temperature in the dark. Controls without primary antibody were also run in parallel. Finally, these sections were examined with an optical microscope (Nikon A1R; Nikon, Tokyo, Japan).

### Western blot

The standard protocol of western blot was performed according to our previous reports.^26,28^ Briefly, the concentration of total protein from segments of small intestine (ileum) was measured with a BCA protein assay kit. 20 µg of protein was fractionated in 12% SDS-polyacrylamide gels and transferred onto polyvinylidene difluoride membranes. The membranes were blocked with 5% freshly prepared milk-TBST for 2 h at room temperature and then incubated overnight at 4°C with the following primary antibodies: anti-Ocln (1:2000; Abcam, Cambridge, USA), anti-Tjp1 (1:1000; Invitrogen, Carlsbad, CA) and anti-Cldn4 (1:3000; Abcam, Cambridge, USA). The membranes were then washed three times for 10 min in TBST and incubated with secondary antibody conjugated with HRP for 2 h. Finally, the membranes were subjected to a chemiluminescence detection system and exposed to a photographic film. Immunoreactsive bands were quantified using Image J software and bands were normalized with β-actin.

### Fecal DNA isolation, library preparation, and bioinformatics

Fecal samples were collected from six to seven animals into sterile tubes and stored at −80°C. The preparation of bacterial 16S rRNA library for MiSeq sequencing was performed as previously reported.^7,21^ DNA was isolated from fecal samples using Zymoresearch Fecal/Soil DNA isolat**i**on kit (Zymoresearch, Irvine, CA). The DNA concentration in samples was determined using NanoDrop One (Thermo Fischer, USA). Illumina compatible primers were used to amplify the bacterial 16S ribosomal DNA V4-V5 regions. Conventional PCR was performed for the amplification products. PCR products were then subjected to gel purification (QIAGEN, Hilden, Germany) prior to quantification by NanoDrop One. Equimolar PCR products were pooled for Miseq library construction and quantified by real-time PCR (Agilent Technologies, USA). Finally, pooled library was run and sequenced on the Illumina Miseq system (Illumina, San Diego, CA).

The QIIME 1.9.1 software was employed to process and analyze the paired-sequence data as previously described.^7,29^ Briefly, the quality of the reads was determined using chimera-picking and quality-filtering. Open reference operational taxonomic units (OTU) were picked and taxonomic assignments using the SILVA database 16S rRNA database (Version 138) were performed to generate OTU table. Additional α-diversity and β-diversity measures were created with QIIME. Differentially significant features at each taxonomic level were identified using linear discriminant analysis with effect size measurements to generate a taxonomic cladogram.

### ELISA studies

Norepinephrine (NE) and intestinal fatty acid-binding protein 2 (I-FABP) in the plasma were measured using a norepinephrine ELISA kit (Abnova, Taiwan) and a I-FABP ELISA kit (R&D Systems, Minneapolis, MN), respectively, per the manufacturer’s guidelines.^26,30,31^ Briefly, blood samples were collected from the abdominal aorta into a blood collection tube and centrifuged immediately at 4000 g for 15 min at 4°C. Enzyme immunoassay for the quantification of NE and I-FABP was performed in duplicate wells according to the manufacturer’s instructions.

### Statistical analysis

Group data were expressed as mean ± SEM and *P*-value <0.05 was considered significant. *P< .05, **P< .01, ***P< .001, and ****P< .0001. Comparisons among groups were assessed using either one-way or two-way ANOVA followed by Tukey’s multiple comparisons post-hoc tests. The values of tail BP were analyzed by repeated-measures ANOVA. The analysis of data and generation of the graph were performed by Graph Pad Prism software (Version 7.0; La Jolla, CA, USA).

## Results

### Effects of exercise training and detraining on blood pressure, cardiac hypertrophy and remodeling of the SHR

The experimental design and the protocol of exercise training are illustrated in Figure 1a. As shown in Figure 1b, the SHR-Sed rats had a significant elevation in the SBP compared with the WKY-Sed rats (SHR-Sed: 199 ± 3 mmHg vs WKY-Sed: 129 ± 3** **mmHg; *P*< .0001). Twelve weeks following exercise training these was a decrease in SBP in SHR-Ex rats compared with the SHR-Sed rats (SHR-Ex: 167 ± 5 mmHg vs SHR-Sed: 199 ± 3** **mmHg; *P*< .0001). Interestingly, after 4 weeks of detraining the SHR-Det rats demonstrated a significantly lowered SBP compared with the SHR-Sed rats (SHR-Det: 177 ± 4 mmHg vs SHR-Sed: 199 ± 3** **mmHg; *P*< .0001). The BP data were confirmed by an intra-arterial mean arterial BP (MAP) recording (Figure 1c). Although both exercise training and detraining reduced the heart rate (HR) in the SHR rats, the effects were not significant (Figure S1A).

In addition, increased heart weight/body weight (HW/BW; *P*< .01; Figure S1B), heart weight/tibia length (HW/TL; *P*< .001; Figure S1C) and left ventricle weight/tibia length (LVW/TL; *P*< .001; Figure S1D) ratios were observed in the SHR-Sed rats compared with those in the WKY-Sed rats, which were ameliorated by exercise training. Interestingly, there was no significant decrease in the HW/BW and LVW/TL ratios in the SHR-Det compared with the SHR-Sed rats although change in the HW/TL ratio was significant. We evaluated general morphology and collagen formation in hearts with H&E and Masson-trichrome staining to determine effects of exercise training and detraining on cardiac hypertrophy and remodeling.

We observed that SHR-Sed rats exhibited a marked increase in the cross sectional area of cardiomyocytes (*P*< .0001; Figure 1d and f) and perivascular fibrosis (*P*< .01; Figure 1e and g) compared with those in the WKY-Sed rats. Interestingly, attenuation of the cross-sectional area of cardiomyocytes (*P*< .01; Figure 1d and f) and perivascular fibrosis (*P*< .01; Figure 1e and g) were observed in the SHR-Ex rats compared with the SHR-Sed rats. However, there was no significant decrease in the cross-sectional area of cardiomyocytes and perivascular fibrosis in the SHR-Det rats compared with the SHR-Sed rats. In contrast, there was no difference in the aforementioned cardiovascular parameters in normotensive rats.

### Exercise training persistently altered gut microbial composition in the SHR

**Figure 2.**
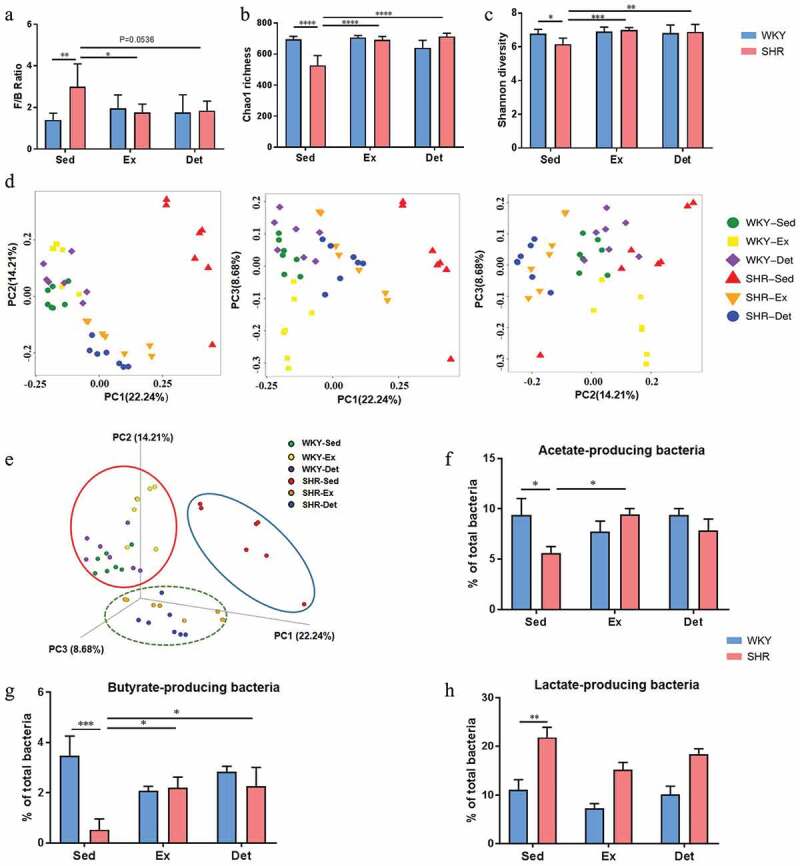
Effects of exercise training and detraining on the remodeling of gut microbiota in SHR and WKY rats. (a) A column graph showing the ratio of phyla Firmicutes to Bacteroidetes (F/B). Chao1 richness (b) and Shannon diversity (c) scores of α-diversity of 16S rRNA sequencing of fecal samples in different groups.Two-dimensional (d) and three-dimensional (e) principal coordinate plot (PCoA) for β-diversity showing the clustering of gut microbial communities in different groups. Column diagrams show acetate- (f), butyrate-(g) and lactate-(h) producing bacteria in different groups. n = 7–8 rats per group. Data are presented as mean ± SEM. *P< .05; **P< .01; ***P< .001; ****P< .0001 using two-way ANOVA with a Tukey’s post-hoc test

Given the vital role of the gut microbiota in multiple models of hypertension,^1,32^ we examine effects of exercise training and detraining on the changes of gut microbiota. The Firmicutes/Bacteroidetes ratio (F/B, an important marker of gut microbiota dysbiosis) was significantly increased in the SHR-Sed rats compared with the WKY-Sed rats (*P*< .01; Figure 2a). Further, the Chao1 richness (*P*< .0001; Figure 2b) and Shannon diversity (*P*< .05; Figure 2c) showed a significant decrease in the SHR-Sed rats. However, exercise training and detraining restored these changes in the SHR. No significant effects of exercise on these parameters were observed in WKY rats. Principal coordinate analysis (PCoA) of unweighted UniFrac distances of the gut microbial composition (Figure 2d and e) demonstrated a clear separation of animal clusters between the WKY-Sed and SHR-Sed group (ANOSIM, *R*= 0.762; *P*= .001), which is consistent with the previously reported observation.^1,21^ Continuous exercise training altered the composition of gut microbial communities in both WKY (ANOSIM, *R*= 0.665; *P*= .001; WKY-Ex versus WKY-Sed) and SHR (ANOSIM, *R*= 0.716; *P*= .002; SHR-Ex versus SHR-Sed). This shift was also observed in the WKY-Det (ANOSIM, *R*= 0.678; *P*= .001; WKY-Det versus WKY-Sed) or SHR-Det (ANOSIM, *R*= 0.714; *P*= .002; SHR-Det versus SHR-Sed) group, indicating a relatively stable gut microbial composition after 4 weeks of detraining (Figure 2d and e). The dysbiosis of gut microbiota in hypertensive animal models is characterized by increased lactate-producing bacteria, and decreased butyrate- and acetate-producing bacteria populations.^4,20^ Table 2 shows the bacteria that were classified as producers of each type of short-chain fatty acids (SCFA).^33^ There was a significant decrease in acetate- (*P*< .05; figure 2f) and butyrate-producing (*P*< .001; Figure 2g) bacterial communities, and an increase in lactate-producing (*P*< .01; Figure 2h) bacterial communities in the SHR-Sed group as compared with the WKY-Sed group. However, these differences were restored by continuous exercise training. Four weeks of detraining resulted in a significant increase in butyrate-producing (*P*< .05; Figure 2g) bacterial communities (especially the changes in the families Lachnospiraceae and Ruminococcaceae; *P*< .05; Figure S2) in the SHR-Det rats compared with the SHR-Sed rats. Moreover, trends toward an increment in acetate-producing bacteria and a reduction in lactate-producing bacteria were observed in the SHR-Det rats.

**Table 2.** Major genera were classified according to the dominant fermentation end-product(s)

Genera	Classification
Ruminococcaceae_UCG-014	A,B
Lactobacillus	L
Ruminococcus_1	A,B
Ruminococcaceae_UCG-005	A,B
Turicibacter	L
Phascolarctobacterium	S
Prevotellaceae_UCG-001	A
Prevotellaceae_NK3B31_group	A
Ruminiclostridium_9	A
[Eubacterium]_coprostanoligenes_group	B
Lachnospiraceae_NK4A136_group	A,B
Prevotella_9	A
Blautia	A
Prevotellaceae_UCG-003	A
Treponema_2	U
Dorea	A
Bacteroides	A
Ruminococcaceae_UCG-013	A,B
Ruminococcaceae_NK4A214_group	A,B
Butyrivibrio	B
Ruminiclostridium_6	A,B
Coprococcus_3	B
Clostridium_sensu_stricto_1	M
Marvinbryantia	A
f__Lachnospiraceae_Unclassified	B
Acetitomaculum	A
[Eubacterium]_xylanophilum_group	B
[Eubacterium]_ruminantium_group	B
Prevotella_1	A
Ruminococcus_2	M
Anaerostipes	B
Parabacteroides	A
Alloprevotella	A,S,B,L
f__Desulfovibrionaceae_Unclassified	U
Ruminococcaceae_UCG-008	A,B
Coprococcus_2	B
f__Peptococcaceae_Unclassified	S,E
Ruminiclostridium_5	A,B
Roseburia	B
Alistipes	S
Intestinimonas	B
Collinsella	M
f__Bacteroidales_RF16_group_Unclassified	A
Ruminococcaceae_UCG-009	B
Lachnoclostridium	B
Parasutterella	U
[Eubacterium]_nodatum_group	B
Ruminiclostridium	A
Allobaculum	U
Lachnospiraceae_ND3007_group	B
Lachnospiraceae_UCG-010	B
Negativibacillus	S,P
Prevotellaceae_Ga6A1_group	A
Ruminococcaceae_UCG-010	A,B
[Eubacterium]_fissicatena_group	B
Bifidobacterium	A,P,B,L
[Eubacterium]_hallii_group	B
Lachnospiraceae_UCG-001	B
Moryella	A,B,L
[Ruminococcus]_gauvreauii_group	A,B
Lachnospiraceae_UCG-006	B
f__Eggerthellaceae_Unclassified	U
Desulfovibrio	A
Lachnospiraceae_NK4B4_group	B
Coprococcus_1	B
[Eubacterium]_ventriosum_group	B
Butyricicoccus	B
Prevotellaceae_UCG-004	A
Akkermansia	U
Lachnospiraceae_FCS020_group	B
Ruminococcaceae_UCG-004	A,B
Lachnospira	A,L,E
f__Christensenellaceae_Unclassified	A
[Bacteroides]_pectinophilus_group	A
Enterorhabdus	U
[Eubacterium]_brachy_group	U
Escherichia-Shigella	B
Subdoligranulum	trace A, S
Barnesiella	L
Lactococcus	U
[Ruminococcus]_torques_group	A,B
Veillonella	P
Streptococcus	L
Butyricimonas	B
Mogibacterium	Phenyl acetate
Rothia	U
Sutterella	S
Odoribacter	A,P,B
Lachnospiraceae_NC2004_group	B
Anaerotruncus	A,B
Anaerofilum	A

A = acetate, B = butyrate, E = ethanol, F = fumarate, M = mixed (assorted C2-C4 compounds), L = lactate, *P* = propionate, S = succinate, U = uncharacterized.

### Exercise training persistently ameliorates gut pathology, inﬂammation and permeability in the SHR

**Figure 3.**
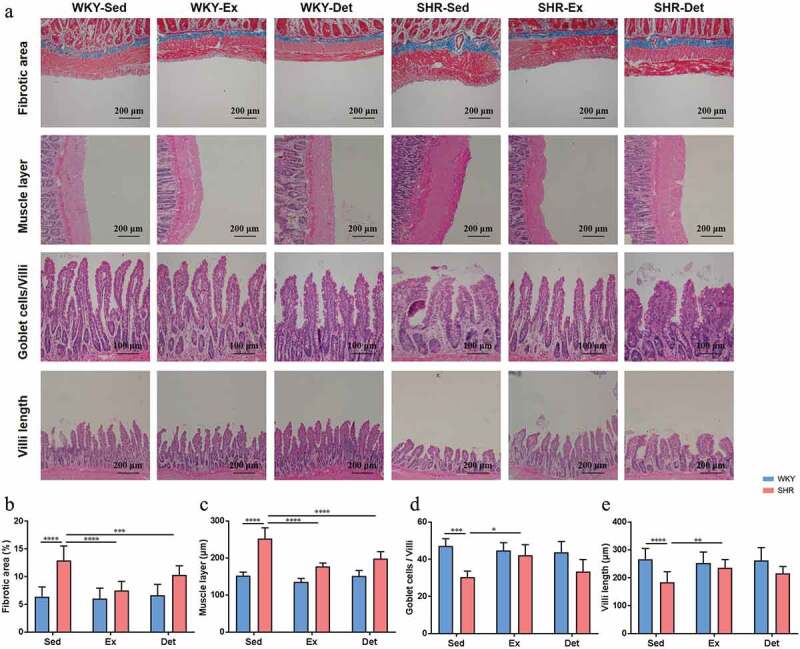
Effects of exercise training and detraining on gut pathological alterations in the ileum in SHR and WKY rats. (a) Representative micrographs of hematoxylin-eosin (H&E) and Masson-trichrome staining assays showing the changes in the ileum in all experimental groups. (b) Cross section staining with Masson-trichrome stain performed to quantify the fibrotic area in the ileum. (c) Cross section staining with H&E stain performed to quantify the thickness of muscle layer in the ileum. (d) Quantitative analysis of cross section stained with H&E stain to observe the ratio of goblet cells/villi in the ileum. (e) Cross section staining with H&E stain to quantify the villi length in the ileum. n = 8–10 rats per group. Data are presented as mean ± SEM. *P< .05; **P< .01; ***P< .001; ****P< .0001 using two-way ANOVA with a Tukey’s post-hoc test

Altered gut pathology, increased gut inﬂammation, and permeability have been demonstrated in the pathogenesis of hypertension.^6,7^ We investigated the effects of exercise training and detraining on gut pathology, inﬂammation and permeability. In the ileum, the SHR-Sed rats exhibited an increase in the fibrotic area (Figure 3a and b) and thickness of the muscle layer (Figure 3a and c) compared with those in the WKY-Sed rats. Furthermore, decreased goblet cells/villi (Figure 3a and d) and villi length (Figure 3a and e) were observed in the ileum of the SHR-Sed rats compared with those in the WKY-Sed rats. Continuous exercise training significantly decreased the fibrotic area and thickness of the muscle layer and increased the goblet cells/villi and villi length in the ileum of the SHR-Ex rats. This trend of beneficial effects on gut pathology was maintained remained even after four weeks of detraining, although increase in goblet cells/villi and villi length did not reach significance (Figure 3). In contrast, no differences in gut pathology were found in the ileum of treatment-matched WKY rats (Figure 3). Consistently, increased fibrotic area (Figure S3A and B) and thickness of muscle layer (Figure S3A and C), and decreased number of goblet cells (Figure S3A and D) and crypt depth (Figure S3A and E) were observed in the proximal colon in the SHR-Sed rats compared to that in the WKY-Sed rats. Continuous exercise training led to a significant reversal in fibrotic area (Figure S3A and B), thickness of muscle layer (Figure S3A and C), and crypt depth (Figure S3A and E) in proximal colon in the SHR-Ex rats, though increase in the number of goblet cells (Figure S3A and D) was not significant. Furthermore, four weeks of detraining persistently ameliorated gut pathology in the SHR-Det rats by decreasing the fibrotic area and thickness of the muscle layer (Figure S3B and C). However, no difference in the number of goblet cells or crypt depth was found between the SHR-Det and SHR-Sed rats (Figure S3D and E). Neither continuous exercise training nor four weeks of detraining altered gut pathology in the proximal colon of WKY rats (Figure S3).

**Figure 4.**
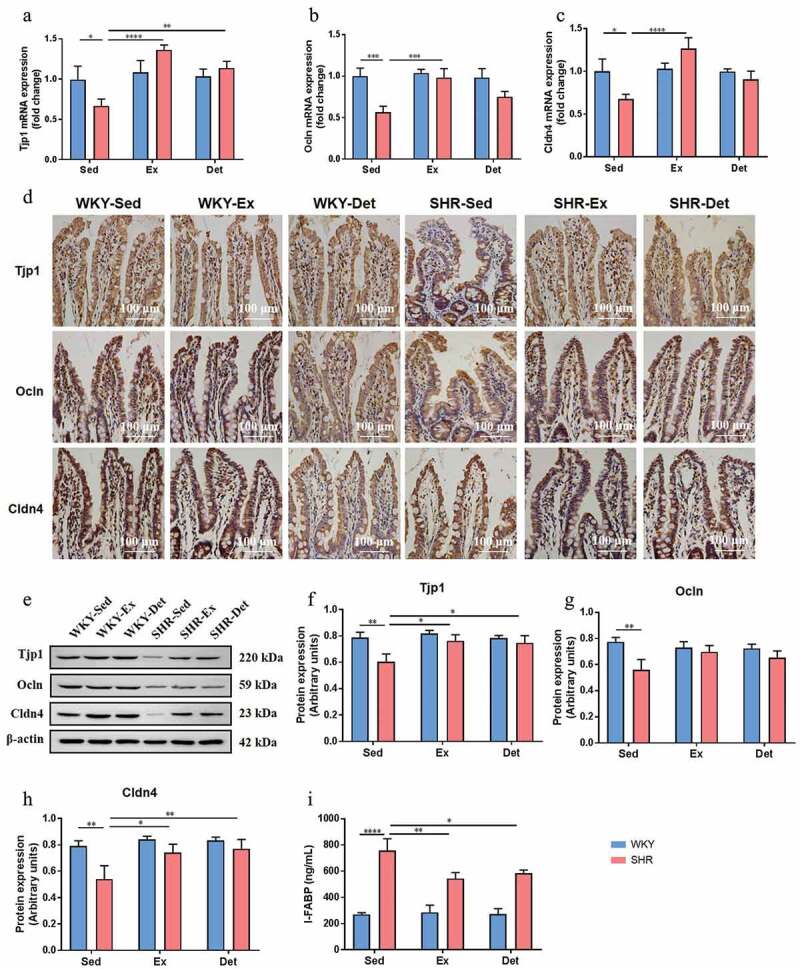
Effects of exercise training and detraining on gut permeability in SHR and WKY rats. The mRNA levels of tight junction proteins Tjp1 (a), Ocln (b) and Cldn4 (c) in small intestine (ileum) in all experimental groups. (d) Representative immunohistochemistry images showing Tjp1-, Ocln- and Cldn4-positive cells in the ileum in SHR and WKY rats. (e) A representative immunoblot; and (f-h) densitometric analysis of protein expression of Tjp1, Ocln and Cldn4 in the ileum in SHR and WKY rats. (i) Measurement of intestinal FABP level in the plasma in SHR and WKY rats. n = 8–10 rats per group. Data are presented as mean ± SEM. *P< .05; **P< .01; ***P< .001; ****P< .0001 using two-way ANOVA with a Tukey’s post-hoc test

Next, we examined mRNA levels of proinﬂammatory cytokines and their receptors (TNF-α, IL-1β, IL-6, HMGB1, TLR2, and TLR4; Figure S4A-F) in the small intestine of the SHR and WKY rats. Increases in their levels were observed in the SHR-Sed rats compared with WKY-Sed rats. Continuous exercise training and detraining attenuated TNF-α (Figure S4A), IL-6 (Figure S4C) and TLR4 (Figure S4F) mRNA levels in the ileum of the SHR-Ex and SHR-Det rats, although changes in the mRNA levels of IL-1β (Figure S4B), HMGB1 (Figure S4D) and TLR2 (Figure S4E) were not significant. In contrast, no differences in mRNA levels of the aforementioned immunity-related genes were observed in the ileum of the treatment-matched WKY rats. Finally, we investigated the effects of exercise training and detraining on gut permeability in the small intestine as measured by changes in the level of tight junction proteins (tight junction protein 1, occludin and claudin 4). As shown in Figure 4a-c, the mRNA levels of Tjp1 (tight junction protein 1), Ocln (occludin) and Cldn4 (claudin 4) were decreased in the ileum of the SHR-Sed compared with the WKY-Sed rats. However, these mRNA levels were restored by continuous exercise training (Figure 4a-c). Importantly, four weeks of detraining persistently increased the Tjp1 mRNA level in the ileum of the SHR-Det rats (Figure 4a). However, increased Ocln and Cldn4 mRNA levels were not significant (Figure 4b and c). In contrast, no difference in the gene expression of the tight junction proteins was observed in the ileum of treatment-matched WKY rats (Figure 4a-c). As shown in Figure 4d, the number of Tjp1-, Ocln- and Cldn4-positive cells was decreased in the ileum of the SHR-Sed compared with the WKY-Sed rats. The western blot results further demonstrated that SHR-Sed rats had a decrease in the protein expression of Tjp1, Ocln and Cldn4 in the ileum in comparison with the WKY-Sed rats. Interestingly, continuous exercise training and detraining increased the immunoreactivity (Figure 4d) and protein levels of Tjp1 (Figure 4e and f), Ocln (Figure 4e and g) and Cldn4 (Figure 4e and h) in the ileum of the SHR, while change in the protein level of Ocln was not significant. Neither continuous exercise training nor 4 weeks of detraining altered the level of tight junction proteins in the ileum of WKY rats (Figure 4).

I-FABP has been used as a marker of gut permeability,^34^ and a growing body of evidence has shown increased circulating I-FABP in animals and human with hypertension.^35,36^ In our study, increased plasma I-FABP was observed in the SHR-Sed rats compared with those in the WKY-Sed rats. Importantly, continuous exercise training and detraining decreased the plasma level of I-FABP in the SHR compared with those in the SHR-Sed rat (Figure 4i).

### Exercise training persistently protects against microglial activation and neuroinﬂammation in the SHR

**Figure 5.**
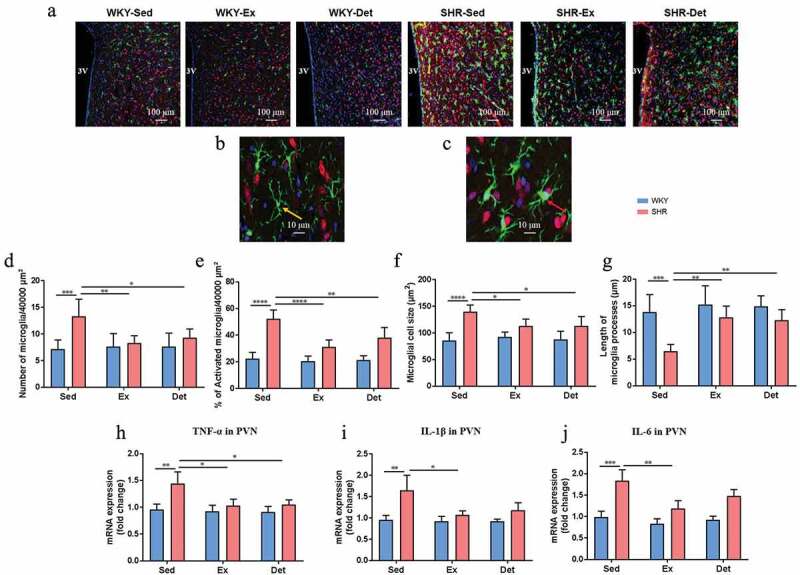
Effects of exercise training and detraining on microglial activation and neuroinﬂammation in SHR and WKY rats. (a) Representative immunofluorescence images at 20× magnification show the paraventricular nucleus (PVN) sections stained with anti-Iba1 (ionized-calcium binding adaptor molecule 1) antibody indicative of microglia (green), anti-NeuN indicative of neurons (red), and DAPI showing DNA (blue). (b) Non-activated microglia exhibits a small cell body (yellow arrow) with thin and highly ramified branches extending in all directions. (c) Activated microglia manifests a more “ameboid” morphology, characterized by larger cell bodies (red arrow) with thickened and shortened processes. (d) Total number of microglia (activated + non-activated) and (e) % of activated microglia within the 40,000 µm^2^ area of PVN. (f) Microglial cell size and (g) average length of microglia processes (n = 15 largest cells per rat) in the PVN. The mRNA levels of TNF-α (h), IL-1β (i) and IL-6 (j) in the PVN measured by real-time PCR and normalized to GAPDH. n = 7–9 rats per group. Data are presented as mean ± SEM. *P< .05; **P< .01; ***P< .001; ****P< .0001 using two-way ANOVA with a Tukey’s post-hoc test

Increased activated microglia and neuroinflammation within autonomic brain regions have been closely associated with established hypertension.^7,21^ We evaluated the changes in microglial cells and neuroinflammation in the PVN (One key autonomic brain region controlling the blood pressure). The total number of microglial cells and the visual characterization of activated microglial cells in the PVN increased significantly in the SHR-Sed rats compared with the WKY-Sed rats (Figure 5a-g). When microglia are activated, they exhibit a larger cell body with well-defined and shorter branches, and become thicker and stubby in appearance.^37,38^ As shown in Figure 5b and c, non-activated microglia show ramified morphology characterized by small cell soma and long, thin processes, while activated microglia exhibit an amoeboid form characterized by large cell soma and short processes. Therefore, we measured the size of the cell and the length of processes in microglia in the PVN under each treatment condition. We found that, compared to the WKY-Sed rats, SHR-Sed rats exhibited an increase in the size of microglial cells (figure 5f) and a decrease in the length of the microglial processes (Figure 5g). Interestingly, continuous exercise training and 4 weeks of the detraining not only decreased the total number of microglia (Figure 5d) and percentage of activated microglia (Figure 5e), but also deceased the microglial cell size (figure 5f) and increased the length of microglial processes (Figure 5g) in the PVN of the SHR-Ex and SHR-Det rats. Previous studies have shown that pro-inflammatory cytokines derived from activated microglia modulate neuronal activity.^39,40^ Next, we measured the levels of proinﬂammatory cytokines in the PVN to determine whether changes in the microglial cells were associated with changes in neuroinﬂammation. Compared with the WKY-Sed rats, SHR-Sed rats exhibited an increase in the mRNA levels of tumor necrosis factor (TNF)-α (*P*< .01; Figure 5h), interleukin (IL)-1β (*P*< .01; Figure 5i) and IL-6 (*P*< .001; Figure 5j), which were attenuated by continuous exercise training. Four weeks of detraining attenuated the increased mRNA levels of pro-inflammatory cytokines in the PVN of SHR-Det rats. However, changes in the level of IL-1β (Figure 5i) and IL-6 (Figure 5j) were not significant. In contrast, no difference between microglial activation and neuroinﬂammation was observed in the PVN of treatment-matched WKY rats (Figure 5).

### FMT decreased the BP and ameliorated cardiac pathology

**Figure 6.**
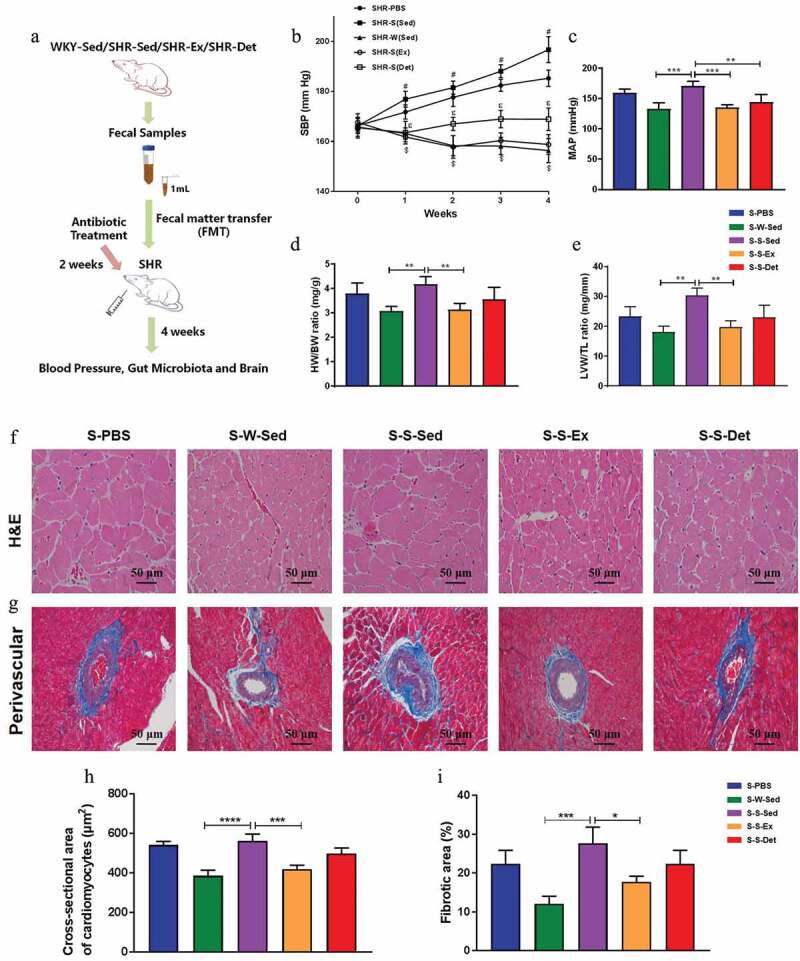
Effects of FMT on blood pressure and heart function in SHR. (a) The experimental protocol of FMT. (b) Time course of SBP was measured by tail-cuff plethysmography under each treatment. (c) MAP was measured by intra-arterial recording in the left carotid artery. Bar graphs show the ratios of HW/BW (d) and LVW/TL (e) under each treatment. (f) Representative micrographs showing the results of H&E staining in terms of cardiomyocytes size in different groups. (g) Representative micrographs of Masson’s trichrome staining assay showing perivascular fibrosis in myocardium. (h) A column diagram showing quantitative analysis of cross-sectional area of cardiomyocytes. (i) A column diagram showing quantitative analysis of the relative fibrotic area. n = 8–11 rats per group. Data are presented as mean ± SEM. SBP data were analyzed by one-way repeated-measures ANOVA with a Tukey’s post-hoc test. ^#^*P*< .05 S-S-Sed versus S-W-Sed; ^$^*P*< .05 S-S-Sed versus S-S-Ex; ^ε^*P*<.05 S-S-Sed versus S-S-Det. Other parameters were analyzed by one-way ANOVA with a Tukey’s post-hoc test. *P< .05; **P< .01; ***P< .001; ****P< .0001

We performed a FMT from the SHR-Exercise into the SHR to confirm the role of the gut microbiota. The experimental protocol of FMT is illustrated in Figure 6a. FMT from the SHR-Sed to the SHR significantly elevated the SBP compared with FMT from the WKY-Sed to the SHR (S-S-Sed: 197 ± 5 mmHg vs S-W-Sed: 156 ± 5** **mmHg; *P*< .0001; Figure 6b). However, after FMT from the SHR-Ex, the SHR showed a significantly lowered SBP (S-S-Ex: 159 ± 4 mmHg vs S-S-Sed: 197 ± 5** **mmHg; *P*< .0001; Figure 6b). FMT from the SHR-Det to the SHR (S-S-Det group) also resulted in a reduced SBP compared with the S-S-Sed group (S-S- Det: 169 ± 4 mmHg vs S-S-Sed: 197 ± 5** **mmHg; *P*< .0001; Figure 6b). Intra-arterial mean blood pressure (MAP) was recorded to confirm the effects of FMT on blood pressure (Figure 6c). Furthermore, the HW/BW (Figure 6d) and LVW/TL (Figure 6e) ratios increased in the S-S-Sed group compared with the S-W-Sed group but were significantly attenuated in the S-S-Ex group. Trends toward decrement of the HW/BW and LVW/TL ratios were observed in the S-S-Det group compared with the S-S-Sed group (Figure 6d and e). To further investigate the FMT effects on the change*s* of cardiac pathology, we evaluated the level of cardiac hypertrophy and remodeling with H&E and Masson-trichrome staining in all experiment groups. We found that S-S-Sed group exhibited a marked increase in the cross-sectional area of cardiomyocytes compared to the S-W-Sed group (figure 6f and h), whereas these changes were ameliorated in the S-S-Ex group (figure 6f and h). Masson’s trichrome staining showed that S-S-Sed group exhibited an increase in perivascular fibrosis (Figure 6g and i), which was attenuated in the S-S-Ex group (Figure 6g and i).

### FMT from the SHR-exercise into the SHR rats reshaped the gut microbiota

**Figure 7.**
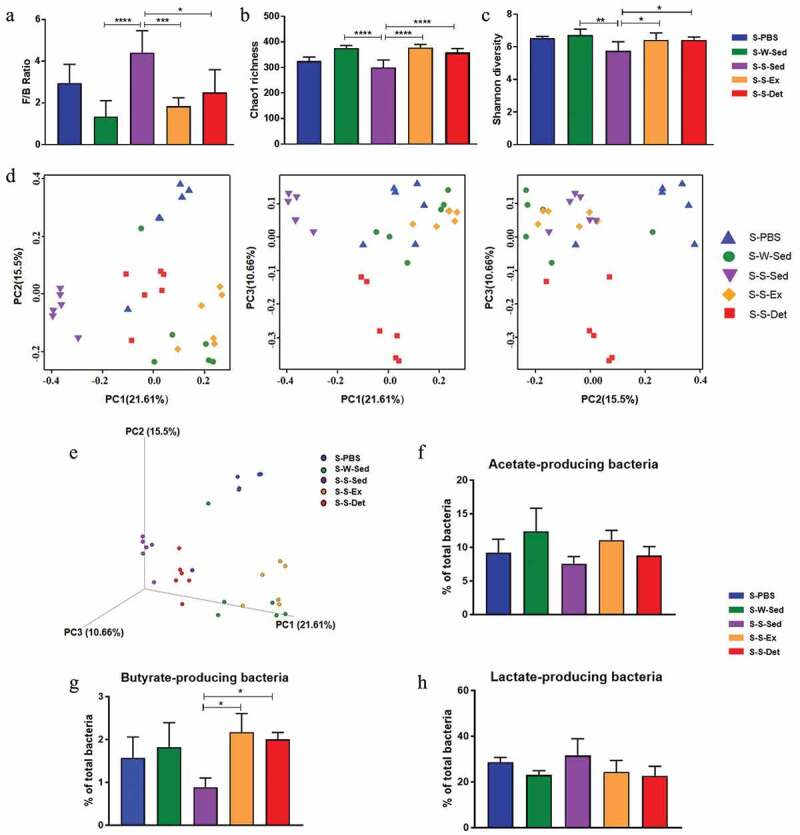
Changes in the composition of gut microbiota after FMT in SHR. Changes in the Firmicutes to Bacteroidetes (F/B) ratio (a), Chao1 richness (b) and Shannon diversity (c) in different groups. Two-dimensional (d) and three-dimensional (e) principal coordinate analysis in the gut microbiota in all experimental groups are shown. The relative proportions of acetate- producing bacteria (f), butyrate- producing bacteria (g) and lactate-producing bacteria (h) in all experimental groups. n = 5–6 rats per group. Data are presented as mean ± SEM. *P< .05; **P< .01; ***P< .001; ****P< .0001using one-way ANOVA with a Tukey’s post-hoc test

The composition of the gut microbiota after FMT was evaluated. As shown in Figure 7 a-c, a significant increase in the F/B (*P* < .0001; Figure 7a) ratio was observed in the S-S-Sed group compared with the S-W-Sed group, which was consistent with previous reports. The major ecological parameters: Chao1 richness (*P* < .0001; Figure 7b) and Shannon diversity (*P* < .01; Figure 7c) were decreased in the S-S-Sed group compared with the S-W-Sed group. Interestingly, these differences were restored by FMT from the SHR treated with exercise training or detraining (Figure 7a-c). In addition, the two- (Figure 7d) and three-dimensional PCoA (Figure 7e) images also demonstrated a clear separation between the S-S-Sed and S-W-Sed groups (ANOSIM, *R*= 0.793; *P*= .004). However, S-S-Ex microbiota cluster was closer to the S-W-Sed group (ANOSIM, *R*= 0.809; *P*= .006; S-S-Ex versus S-W-Sed) than the S-S-Sed group (ANOSIM, *R*= 1; *P*= .003; S-S-Ex versus S-S-Sed), and S-S-Det microbiota cluster was located in the middle of the first few groups, indicating an alteration of the gut microbial composition.

Figure S5A showed the relative composition of the five most abundant bacteria at the phylum level in the fecal samples obtained from all experimental groups. Phylum level analysis showed increased Firmicutes (*P*< .0001) and Patescibacteria, and decreased Bacteroidetes (*P*< .0001), Proteobacteria and Actinobacteria in the S-S-Sed group compared with S-W-Sed group (Figure S5B). FMT from the SHR-Ex or the SHR-Det into the SHR resulted in decreased Firmicutes and increased Bacteroidetes, although changes in the level of Proteobacteria, Patescibacteria and Actinobacteria were not significant (Figure S5B). Patescibacteria is a major radiation of candidate phyla. Patescibacteria have small genomes and a presumed symbiotic or parasitic lifestyle, but the difficulty in culturing representative members constrains the characterization of behavioral and adaptive traits.^41^ In addition, an increased proportion of lactate-producing bacteria and a decreased proportion of acetate- and butyrate-producing bacteria were observed in the S-S-Sed compared with S-W-Sed group (figure 7f-h). We found a significant increase of butyrate-producing bacteria after 4 weeks of FMT from the SHR-Exercise or the SHR-detraining into the SHR (Figure 7g). In the cladogram (Figure S6A and Figure S6B), the family Lachnospiraceae and Ruminococcaceae, and the genera Eubacterium_xylanophilum_group, Lachnospiraceae_ND3007_group, Roseburia, Ruminococcaceae_UCG-005 and Ruminiclostridium_6 were enriched in the S-W-Sed compared with S-S-Sed group. The genera in the Lachnospiraceae family (ie, Eubacterium_xylanophilum_group, Lachnospiraceae_ND3007_group and Roseburia) and the genera in the Ruminococcaceae family (i.e., Ruminococcaceae_UCG-005 and Ruminiclostridium_6) in the S-S-Det group were higher than those in the S-S-Sed group (Figure S6B). The aforementioned bacteria enriched in the S-S-Det group were also induced in the S-S-Ex group compared with the S-S-Sed group, although the average abundance was not as high as that in the S-S-Det group (Figure S6B).

### FMT from the SHR-exercise into the SHR rats decreased microglial activation and neuroinﬂammation in the PVN

**Figure 8.**
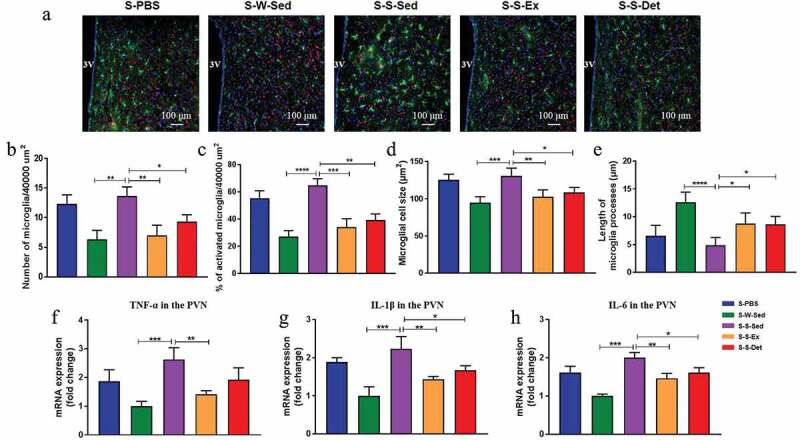
Changes in the activated microglia and neuroinﬂammation of PVN after FMT in SHR. (a) The upper pictures show the number of microglia with anti-Iba1 antibody indicative of microglia (green), anti-NeuN indicative of neurons (red), and DAPI showing DNA (blue). (b) Total number of microglia (activated + non-activated) and (c) % of activated microglia within the 40,000 µm^2^ area of PVN. (d) Microglial cell size and (e) average length of microglia processes (n = 15 largest cells per rat) in the PVN. PVN mRNA levels of TNF-α (f), IL-1β (g) and IL-6 (h) measured under each treatment. n = 8–11 rats per group. Data are presented as mean ± SEM. *P< .05; **P< .01; ***P< .001; ****P< .0001 using one-way ANOVA with a Tukey’s post-hoc test

We observed changes in microglia activation and neuroinﬂammation in the PVN after FMT to confirm the role of gut microbiota in autonomic brain regions. A significant increase in the number (Figure 8a and b) and a percentage of activated microglial cells (Figure 8a and c) was observed in the PVN of the S-S-Sed group compared with the S-W-Sed group. Meanwhile, we observed that S-S-Sed group exhibited an increase in the microglial cell size (Figure 8d) and a decrease in the length of microglial processes (Figure 8e) in the PVN compared to the S-W-Sed group. Interestingly, FMT from the SHR-Ex or the SHR-Det into the SHR resulted in not only a significant reduction in the number and percentage of activated microglial cells (Figure 8a-c), but also a decrease in the microglial cell size (Figure 8d) and an increase in the length of microglial processes (Figure 8e) in the PVN. Furthermore, the mRNA levels of TNF-α (figure 8f), IL-1β (Figure 8g) and IL-6 (Figure 8h) were significantly higher in the PVN of the S-S-Sed rats than the S-W-Sed rats. We found a significant decrease in mRNA levels of TNF-α, IL-1β and IL-6 in the PVN during FMT from the SHR-Ex or the SHR-Det into the SHR (figure 8f-h).

### FMT from the SHR-exercise into the SHR attenuates neuron activity in the PVN

**Figure 9.**
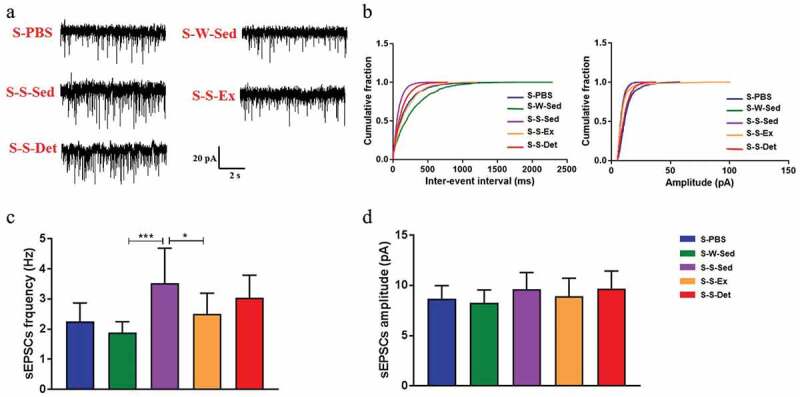
Changes in the physiological properties of PVN neurons after FMT in SHR. (a) Representative traces showing the spontaneous excitatory postsynaptic currents (sEPSCs) recorded in the PVN neurons in all experimental groups. (b) Cumulative inter-event interval (left) and amplitude histograms of the sEPSCs under each treatment. Statistical results of frequency (c) and amplitude (d) of the sEPSCs (n = 15 neurons/5 rats) in all experimental groups. Data are presented as mean ± SEM. *P< .05; **P< .01; ***P< .001; ****P< .0001 using one-way ANOVA with a Tukey’s post-hoc test

Previous studies also found enhanced gut-neuronal communication in animals with hypertension, which originates from paraventricular nucleus of the hypothalamus and presents an increased sympathetic activity in the gut.^6,21^ To determine the physiological properties of neural cardiovascular control center after FMT, spontaneous EPSCs (sEPSCs) were recorded in the PVN neurons. A representative traces of the sEPSCs in the PVN neurons is illustrated in Figure 9a. As shown in Figure 9b-d, the frequency of sEPSCs was significantly higher in the S-S-Sed group compared with the S-W-Sed group but with no statistical significance in changes in the amplitude. An improvement in spontaneous EPSCs was observed in FMT from SHR treated with exercise training. Trends toward a decrement in spontaneous EPSCs were also found in the S-S-Det group.

### FMT from the SHR-exercise into the SHR attenuates the sympathetic activity

**Figure 10.**
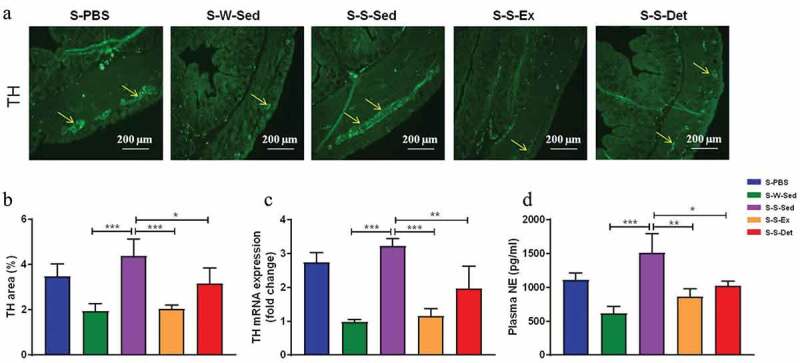
Changes in the sympathetic activity after FMT in SHR. (a) Representative immunofluorescence images of tyrosine hydroxylase (TH) in the proximal colon in all experimental groups. (b) A column diagram showing the statistical results of TH in the proximal colon under each treatment. (c) The mRNA level of TH measured by real-time RT-PCR in all experimental groups. (d) A column diagram showing plasma norepinephrine (NE) level under each treatment. n = 8–11 rats per group. Data are presented as mean ± SEM. *P< .05; **P< .01; ***P< .001; ****P< .0001 using one-way ANOVA with a Tukey’s post-hoc test

The excessive sympathetic activity contributes to the pathogenesis of hypertension and progression of organ damage.^42^ Norepinephrine (NE) and tyrosine hydroxylase (TH; a key enzyme in norepinephrine generation) are usually used as indirect indexes of sympathetic activity.^43^ To test changes of sympathetic activity after FMT, the level of TH in the proximal colon and NE in the plasma were measured. An increase in the TH ﬂuorescence intensity (*P* < .001; Figure 10A and b) and the TH mRNA level (*P* < .001; Figure 10C) was observed in the small intestine of the S-S-Sed rats compared with the S-W-Sed rats. Further, the S-S-Sed rats exhibited a significant increase in the plasma NE level compared with the S-W-Sed rats (*P* < .01; Figure 10D). These differences were restored by FMT from the SHR treated with exercise training or detraining (Figure 10).

## Discussion

The most significant observation of this study is that persistent antihypertensive effect of exercise in the SHR is associated with rebalancing of the dysfunctional gut-brain axis. Evidence for this include: (i) attenuation of gut wall pathology and leakiness; (ii) repopulation with bacterial communities linked with normal BP; (iii) attenuation of PVN microglia and inflammatory markers and neuronal activity; (iv) dampened sympathetic activity and (v) FMT from exercised SHR ameliorates HTN-linked impaired gut-brain axis. Thus, beneficial effects of exercise could, in part, be to influence the gut-brain axis.

Accumulating evidence suggests that gut microbiota and gut-brain communication are crucial in the initiation, development, and establishment of hypertension.^1,2,44^ Moreover, a direct link between gut dysbiosis and hypertension has been confirmed in patients and animal models with hypertension.^3,35,45^ The main characteristics of gut dysbiosis in hypertensive animal models were: (a) gut microbiota is less rich and less diverse, and shows an increased F/B ratio; (b) increased lactate-producing bacteria, and decreased butyrate- and acetate-producing bacteria populations.^4,20^ Exercise training influences the gut microbial composition and lowers BP.^13,16,18,46,47^ Exercise training also increases the proportion of short chain-fatty acids (SCFA)-producing genera in the Firmicutes phylum.^16,18^ Previous studies indicated that the beneficial effect of exercise training on the BP is maintained in angiotensin II–induced hypertensive rats even after withdrawal from exercise training.^12^ However, the pathways involved and the mechanisms of prolonged antihypertensive influence have remained largely speculative. Thus, the present study addressed this gap and demonstrated that exercise-induced alteration of gut microbiota and amelioration of the dysfunctional gut-brain communication was partially responsible for exercise-induced prolonged effects on HTN pathophysiology.

In our study, we found that moderate-intensity exercise in the SHR, even with four weeks of detraining, increased microbial α diversity, altered β diversity, and promoted the growth of butyrate-producing bacteria in the families Lachnospiraceae and Ruminococcaceae of the Firmicutes phylum. Ruminococcaceae and Lachnospiraceae are two major microbial families in the Firmicutes phylum,^48,49^ which are involved in energy extraction from the diet and the fermentation of carbohydrates to produce butyrate. They decrease in hypertensive animal and humans.^35,50^ A reduction in butyrate-producing bacteria leads to a decrease in butyrate level, implicating hypertension pathology. This is consistent with the observations that butyrate is associated with reduced MAP in angiotensin II–induced hypertensive animals,^35^ and it also produces protective effects on neuroinflammation^51^ and induces gut antibacterial peptides to counteract inflammatory and infectious processes.^52,53^ Evidence has demonstrated that impaired intestinal barrier integrity is associated with the pathogenesis of hypertension.^6^ This is supported by recent studies in SHR and angiotensin II–induced hypertensive animal models.^6,7^ The impacts of exercise on the gut have been controversial, presumably due to the intensity of exercise. Extreme exercise in humans causes gut disturbances and increased intestinal permeability.^54,55^ Moderate-intensity exercise shows beneficial impacts on the gut.^16,56^ Our current study favors the latter point: (i) moderate-intensity exercise protects against the impairment of intestinal barrier while normalizing the blood pressure in SHR; (ii) moderate-intensity exercise exerts prolonged beneficial effects on the intestinal pathologies and inflammation. In addition, a significant increase in microglial activation and neuroinflammation in the PVN was attenuated by moderate-intensity exercise training. These changes were maintained in the SHR even after withdrawal from exercise training. Taken together, all exercise-induced alterations in the microbiota-gut-brain axis were associated with a reduced BP and ameliorated cardiac pathology.

In our current study, persistently lowered BP and attenuation of the HTN pathology by exercise training could be linked to a rebalance in the gut-brain axis as a result of altered host-microbiota crosstalk. This view is strongly supported by our FMT experiments. They show that FMT from exercised SHR, which maintain lower BP, into SHR attenuates neuronal activity and neuroinflammation, alters gut microbiota and decreases in SBP. This suggests that exercise rebalances gut microbiota and ameliorates impaired gut-brain axis to mediate antihypertensive effects.

Interestingly, FMT from the SHR-Ex/SHR-Det into the SHR resulted in a decreased F/B ratio, increased microbial α diversity and altered β diversity. This also enriched butyrate-producing bacterial communities. These results are consistent with previous evidence that FMT from the WKY to the SHR decreased the Firmicutes/Bacteroidetes ratio compared with FMT from the SHR to the SHR rats,^4^ which was due to a significant reduction in the Firmicutes abundance. Consistent with exercising experiment, we observed increased butyrate-producing bacteria (e.g. Lachnospiraceae and Ruminococcaceae) in the SHR following transplantation of the fecal microbiota from exercising rats. Taken together, a reduction in BP and an improvement of HTN pathology by moderate-intensity exercise appears to be, at least partially, attributed to the positive alteration in the gut microbiota (especially the changes in butyrate-producing bacteria).

Our previous studies have led to the proposal established that an activated microglia-neuron unit in the PVN increases gut sympathetic drive; this is associated with increased gut pathology and inflammatory status, and altered gut microbiota and permeability.^6,57^ Similarly, recent evidence demonstrated the existence of a brain-gut communication driven by a sympathetic nervous system.^5,7^ Inhibition of microglial activation and neuroinflammation by intracerebroventricular administration of a modified tetracycline normalized sympathetic activity, and attenuated increased mean arterial pressure and left ventricular hypertrophy. This was associated with profound changes in the composition of the gut microbiota and amelioration of gut pathology.^7^ In this study, FMT from the SHR-Ex/SHR-Det into the SHR decreased microglial activation and the excitability of neurons in the PVN and attenuated sympathetic drive. Furthermore, the SHR after FMT from the SHR-Ex/SHR-Det demonstrated a significantly lowered BP and attenuation of cardiac pathology. Our results enrich the view of gut-brain communication in BP control and HTN. Evidence from this study indicates that well-rebalanced gut microbiota and an improved dysfunctional gut-brain axis may be a novel mechanism of exercise-induced blood-pressure-lowering effects.

**Figure 11.**
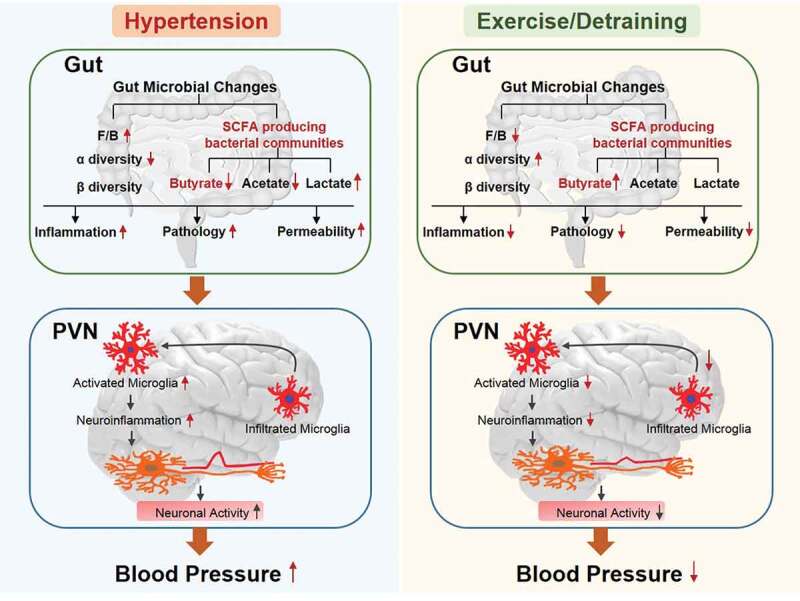
A schematic depicting the proposed pathways of effects of moderate- intensity exercise training and detraining on genetic hypertension. The left panel displays a dysfunctional gut-brain axis in hypertension and the right panel displays that moderate-intensity exercise in the SHR (spontaneously hypertensive rats), even with four weeks of detraining, produces a long-term antihypertensive effect and rebalanced dysfunctional gut-brain axis in hypertensive rats. F/B, Firmicutes/Bacteroidetes ratio (an important marker of gut microbiota dysbiosis); SCFA, short-chain fatty acids; PVN, paraventricular nucleus

In conclusion, our results demonstrate that exercise training, even after its withdrawal, reduces high BP and ameliorates cardiac pathology in rats with genetic hypertension. These effects are associated with changes in the gut microbiota, improvement of gut pathology and permeability, and decreased microglial activation. Additionally, FMT from the SHR-Exercise donor rats into the recipient rats rebalances gut microbial communities, promotes the growth of butyrate-producing bacteria, attenuates activation of microglia, decreases paraventricular nucleus neuronal activity and protects against HTN. Overall, persistently decreased BP and improved HTN pathology are partially attributed to exercise-induced sustained improvement in the microbiome-gut-brain axis (Figure 11). However, further investigation is needed to verify the precise mechanisms of exercise-induced changes in gut microbiota and gut-brain communication.

## Supplementary Material

Supplemental Material
