# Supplementary material for: Antihypertensive effects of exercise involve reshaping of gut microbiota and improvement of gut-brain axis in spontaneously hypertensive rat
Source: Gut Microbes. 2020 Dec 31;13(1):1–24. doi: 10.1080/19490976.2020.1854642 (PMC7781639; doi:10.1080/19490976.2020.1854642)
Supplement: Supplemental Material [file KGMI_A_1854642_SM7888.doc]

Antihypertensive effects of exercise involve reshaping of gut microbiota and improvement of gut-brain axis in spontaneously hypertensive rat

Wen-Jie Xia^a,1^, Meng-Lu Xu^b,1^, Xiao-Jing Yu^a,1^, Meng-Meng Du^a^, Xu-Hui Li^c^, Tao Yang^d^, Lu Li^b^, Ying Li^a^, Kai B. Kang^e^, Qing Su^a^, Jia-Xi Xu^a^, Xiao-Lian Shi^f^, Xiao-Min Wang^a^, Hong-Bao Li^a^,*, Yu-Ming Kang^a^,*

^a^ Department of Physiology and Pathophysiology, Xi'an Jiaotong University School of Basic Medical Sciences, Shaanxi Engineering and Research Center of Vaccine, Key Laboratory of Environment and Genes Related to Diseases of Education Ministry of China, Xi'an 710061, China

^b^ Department of Nephrology, the First Affiliated Hospital of Xi'an Medical University, Xi'an 710003, China

^c^ Center for Neuron and Disease, Frontier Institutes of Science and Technology, Xi’an Jiaotong University, Xi’an 710049, China

^d^ Microbiome Consortium and Center for Hypertension and Precision Medicine, Department of Physiology and Pharmacology, College of Medicine and Life Sciences, University of Toledo, Toledo, OH 43614, USA

^e^ Department of Ophthalmology and Visual Sciences, University of Illinois at Chicago, Chicago, IL

^f^ Department of Pharmacology, School of Basic Medical Sciences, Xi'an Jiaotong University Health Science Center, Xi'an 710061, China

Short Title: Exercise, hypertension and gut-brain axis

* Corresponding author: Hong-Bao Li or Yu-Ming Kang, M.D., Ph.D.

Department of Physiology and Pathophysiology, Xi'an Jiaotong University School of Basic Medical Sciences; Key Laboratory of Environment and Genes Related to Diseases of Ministry of Education; Xi'an Jiaotong University, Xi'an 710061, China.

Xi’an 710061, China

Phone: +86 2982657677

Fax: +86 2982657677

[hongbaoli1985@163.com](mailto:hongbaoli1985@163.com) or [ykang@mail.xjtu.edu.cn](mailto:ykang@mail.xjtu.edu.cn)

**
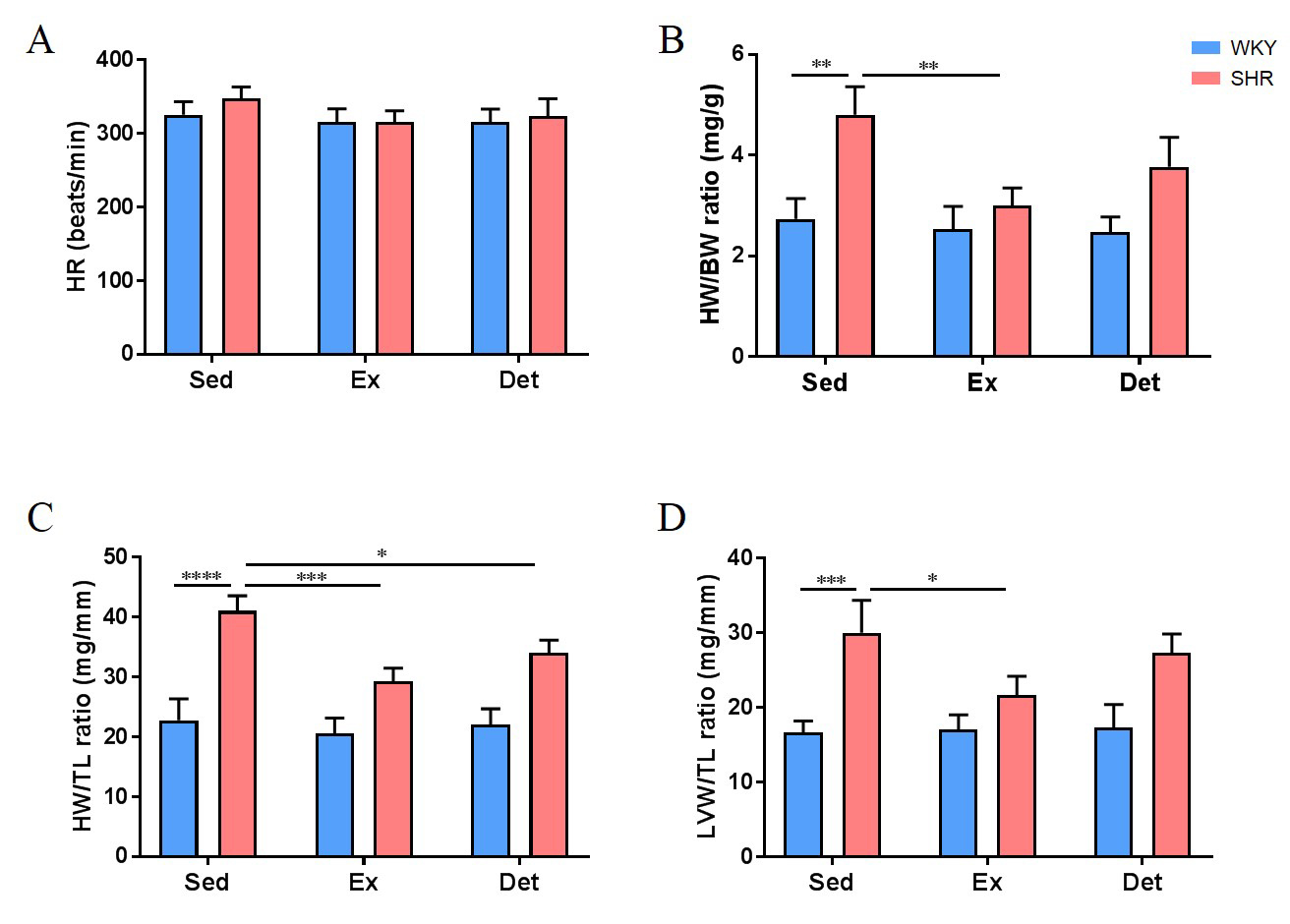
**

**Figure S1.** Effects of exercise training and detraining on HR, HW/BW, HW/TL and LVW/TL in SHR and WKY rats. (A) Changes in heart rate (HR) in different groups. (B) Bar graphs show the ratios of heart weight to body weight (HW/BW), (C) heart weight/tibia length (HW/TL) and (D) left ventricle weight/tibia length (LVW/TL) in different groups. n= 8-10 rats per group. Data are presented as mean ± SEM. ^*^*P*<0.05; ^**^*P*<0.01; ^***^*P*<0.001; ^****^*P*<0.0001 using two-way ANOVA with a Tukey’s post-hoc test.


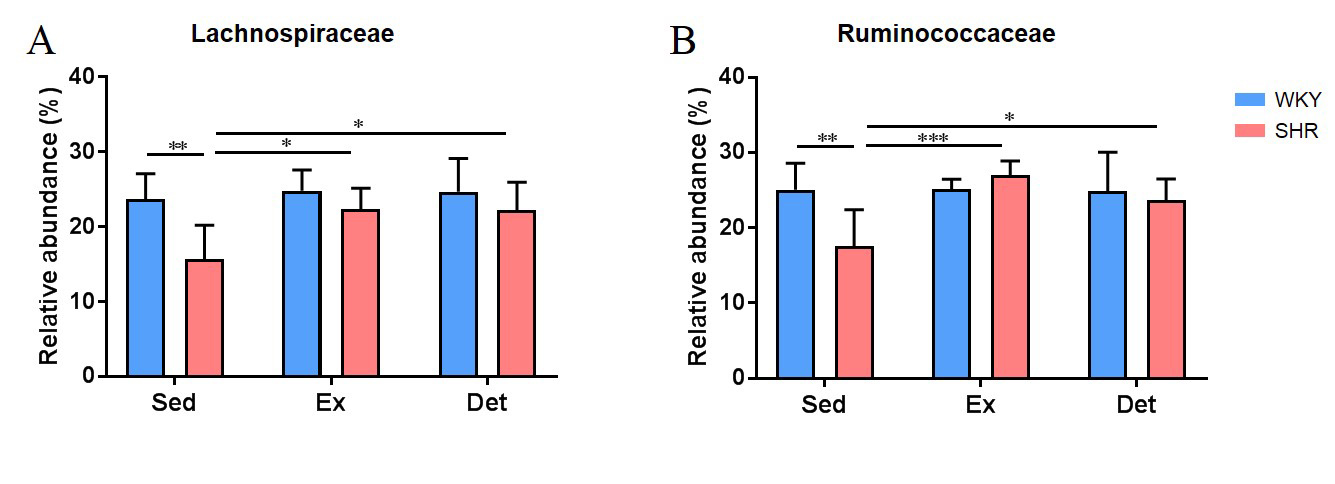


**Figure S2.** Effects of exercise training and detraining on the changes of family Lachnospiraceae and Ruminococcaceae in SHR and WKY rats. (A) The relative abundance of Lachnospiraceae in different groups. (B) The relative abundance of Ruminococcaceae in different groups. n= 7-8 rats per group. Data are presented as mean ± SEM. ^*^*P*<0.05; ^**^*P*<0.01; ^***^*P*<0.001; ^****^*P*<0.0001 using two-way ANOVA with a Tukey’s post-hoc test.

**
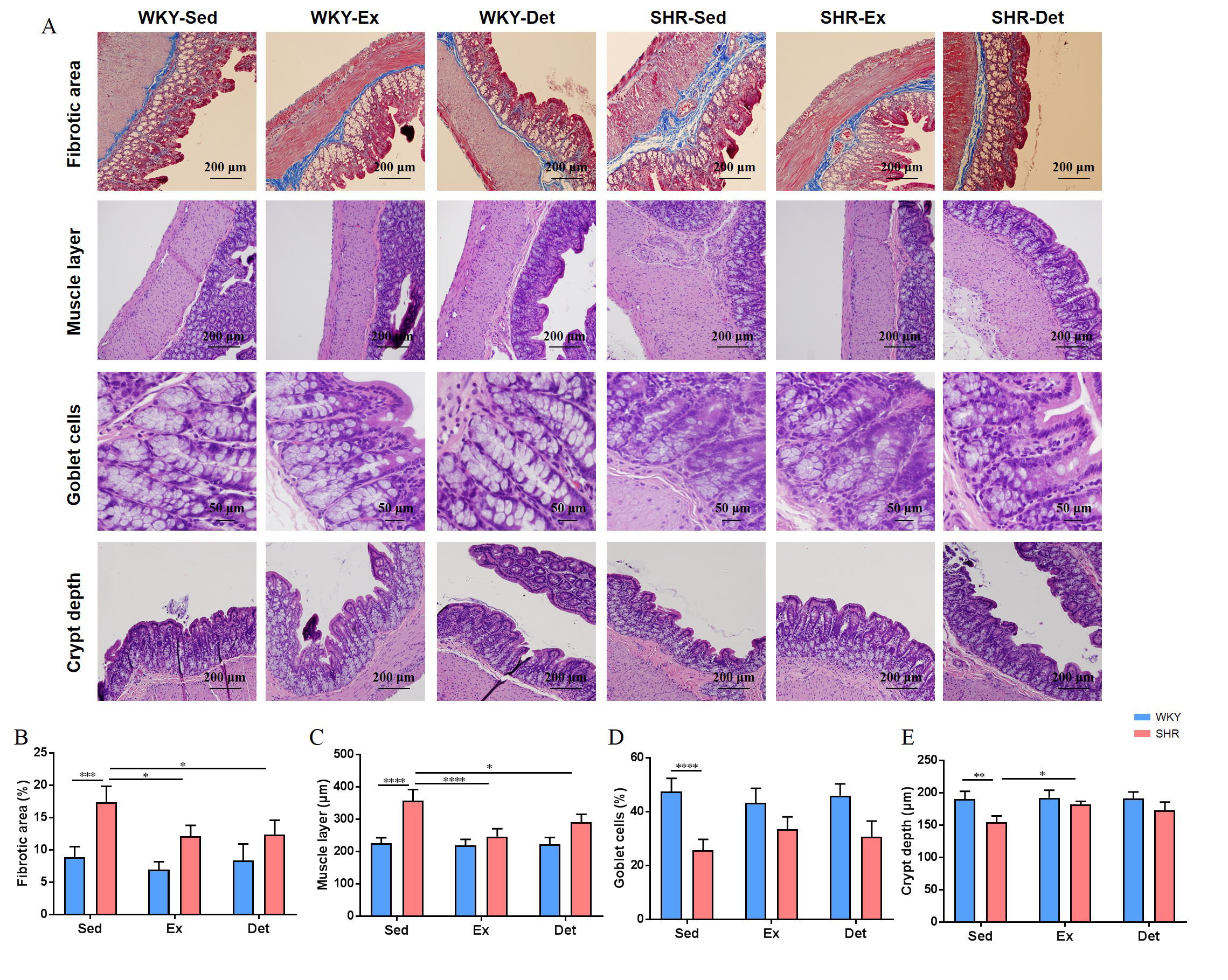
**

**Figure S3.** Effects of exercise training and detraining on gut pathological alterations the proximal colon in SHR and WKY rats. (A) Representative micrographs of hematoxylin-eosin (H&E) and Masson-trichrome staining assays showing the changes in the proximal colon in all experimental groups. (B) Cross section Masson-trichrome staining performed to quantify the fibrotic area in proximal colon. (C) Cross section staining with H&E stain performed to quantify the thickness of muscle layer in the proximal colon. (D) Quantitative analysis of cross section stained with H&E stain to observe the number of goblet cells per 100 epithelial cells in the proximal colon. (E) Cross section staining with H&E stain to quantify the crypt depth in the proximal colon. n= 8-10 rats per group. Data are presented as mean ± SEM. ^*^*P*<0.05; ^**^*P*<0.01; ^***^*P*<0.001; ^****^*P*<0.0001 using two-way ANOVA with a Tukey’s post-hoc test.


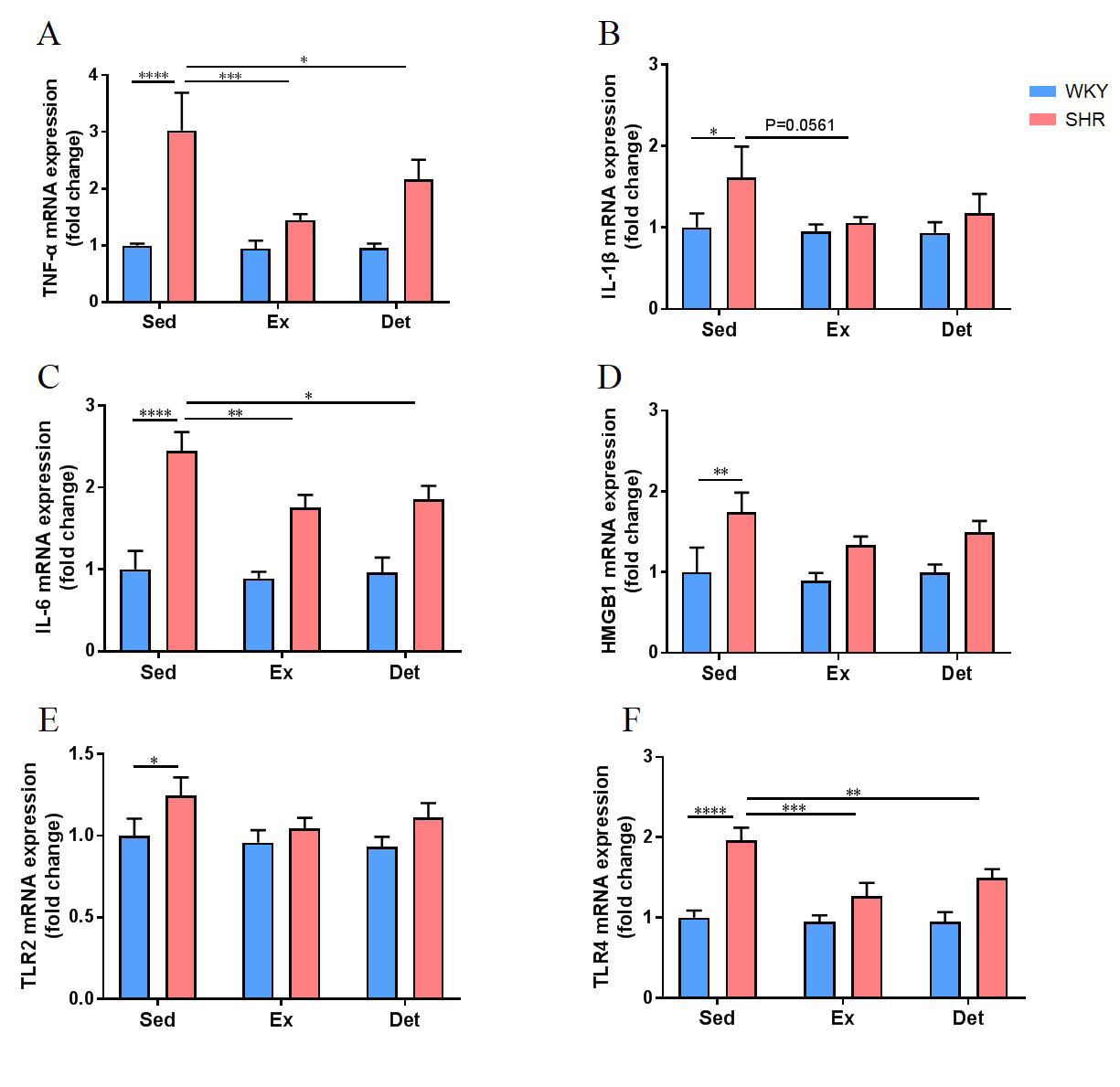


**Figure S4.** Effects of exercise training and detraining on gut inﬂammatory in SHR and WKY rats. The mRNA levels of proinflammatory cytokines TNF-α (A), IL-1β (B) and IL-6 (C), and their receptors HMGB1 (D), TLR2 (E) and TLR4 (F) in small intestine (ileum) in all experimental groups. n= 8-10 rats per group. Data are presented as mean ± SEM. ^*^*P*<0.05; ^**^*P*<0.01; ^***^*P*<0.001; ^****^*P*<0.0001 using two-way ANOVA with a Tukey’s post-hoc test.

**
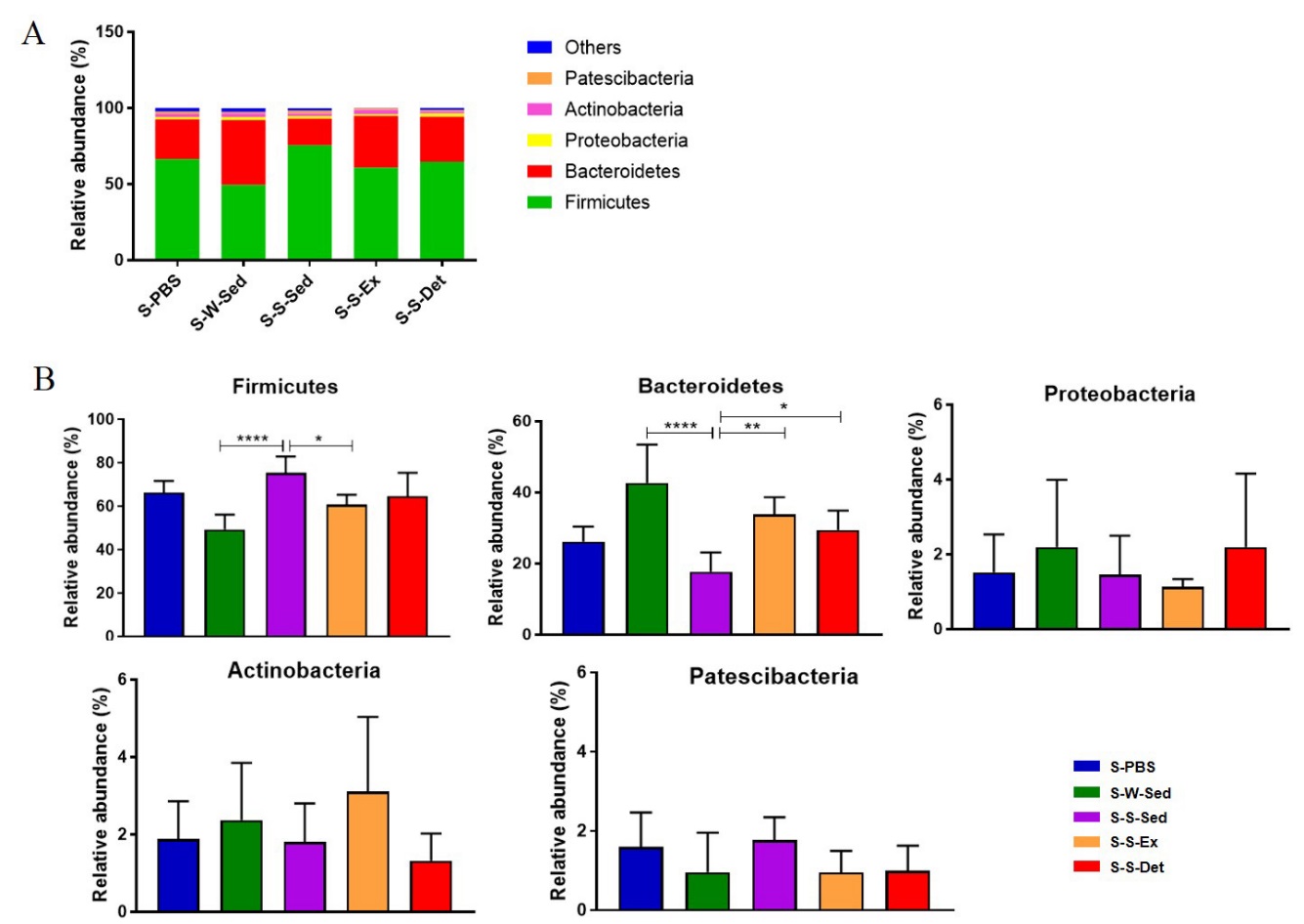
**

**Figure S5.** Changes in the composition of gut microbiota in the phylum level after FMT in SHR. (A) Comparison of phylum-level proportional abundance of fecal samples under each treatment. (B) Bar graphs show individual phylum Firmicutes, Bacteroidetes, Proteobacteria, Actinobacteria and Patescibacteria in different groups. n= 5-6 rats per group. Data are presented as mean ± SEM. ^*^*P*<0.05; ^**^*P*<0.01; ^***^*P*<0.001; ^****^*P*<0.0001using one-way ANOVA with a Tukey’s post-hoc test.

**
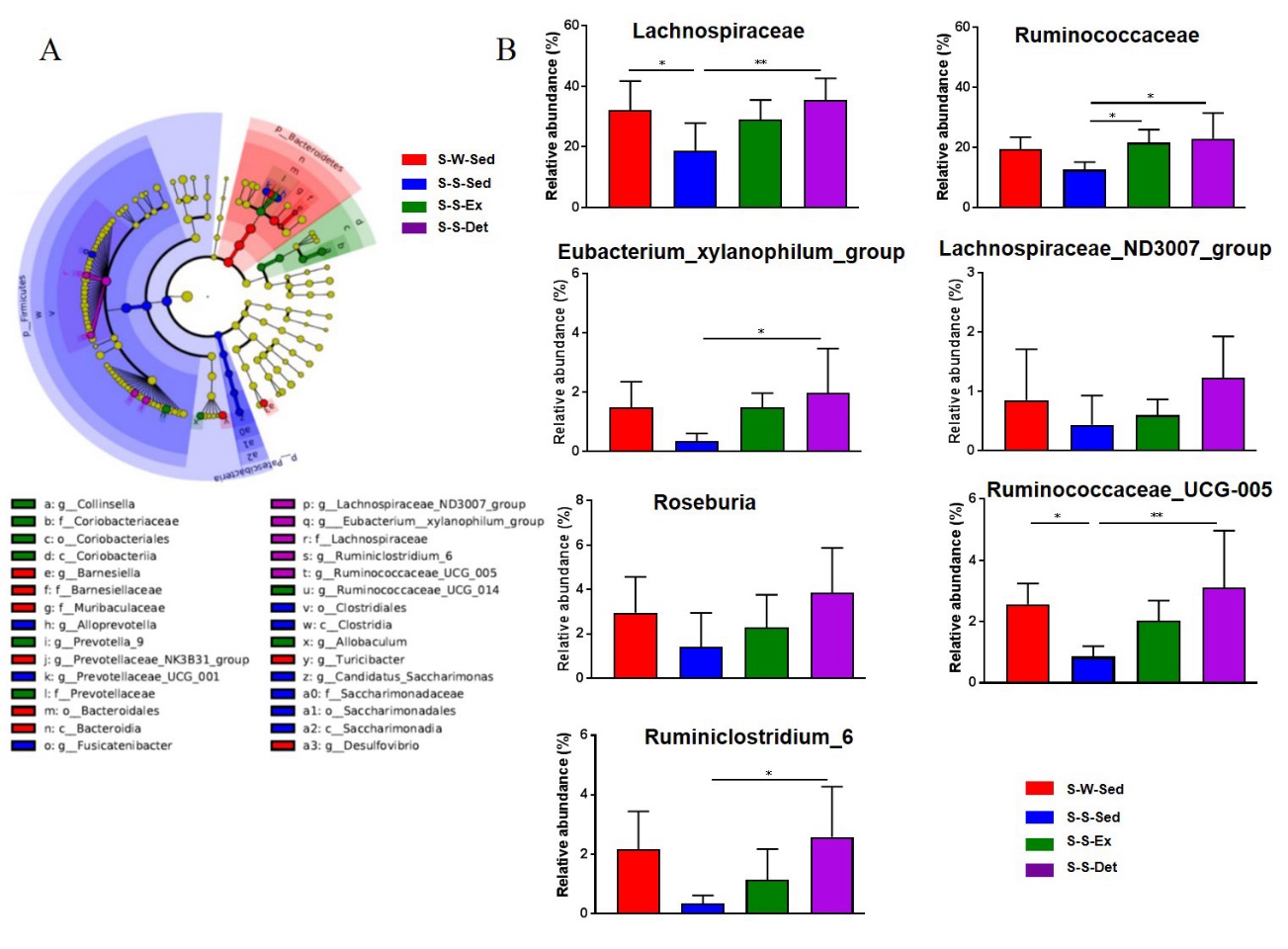
**

**Figure S6.** Changes in the composition of gut microbiota after FMT in SHR. (A) Taxonomic structure and abundance of indicated phylotypes were analyzed by LefSe and presented in cladogram. Significant dominance of phylotypes in each group was indicated by color. (B) Abundance of individual bacterial genera with significant differences between the groups. n= 5-6 rats per group. Data are presented as mean ± SEM. ^*^*P*<0.05; ^**^*P*<0.01; ^***^*P*<0.001; ^****^*P*<0.0001using one-way ANOVA with a Tukey’s post-hoc test.
